# A Randomised Controlled Trial of Ion-Exchange Water Softeners for the Treatment of Eczema in Children

**DOI:** 10.1371/journal.pmed.1000395

**Published:** 2011-02-15

**Authors:** Kim S. Thomas, Tara Dean, Caroline O'Leary, Tracey H. Sach, Karin Koller, Anthony Frost, Hywel C. Williams, the SWET Trial Team

**Affiliations:** 1 Centre of Evidence Based Dermatology, University of Nottingham, Nottingham, United Kingdom; 2 School of Health Sciences and Social Work, University of Portsmouth, Portsmouth, United Kingdom; 3 Medical Research Council Clinical Trials Unit, London, United Kingdom; 4 Medicine, Health Policy and Practice, University of East Anglia, Norwich, United Kingdom; 5 UK Water Treatment Association, Loughborough, United Kingdom; Edinburgh University, United Kingdom

## Abstract

**Background:**

Epidemiological studies and anecdotal reports suggest a possible link between household use of hard water and atopic eczema. We sought to test whether installation of an ion-exchange water softener in the home can improve eczema in children.

**Methods and Findings:**

This was an observer-blind randomised trial involving 336 children (aged 6 months to 16 years) with moderate/severe atopic eczema. All lived in hard water areas (≥200 mg/l calcium carbonate). Participants were randomised to either installation of an ion-exchange water softener plus usual eczema care, or usual eczema care alone. The primary outcome was change in eczema severity (Six Area Six Sign Atopic Dermatitis Score, SASSAD) at 12 weeks, measured by research nurses who were blinded to treatment allocation. Analysis was based on the intent-to-treat population. Eczema severity improved for both groups during the trial. The mean change in SASSAD at 12 weeks was −5.0 (20% improvement) for the water softener group and −5.7 (22% improvement) for the usual care group (mean difference 0.66, 95% confidence interval −1.37 to 2.69, *p* = 0.53). No between-group differences were noted in the use of topical corticosteroids or calcineurin inhibitors.

**Conclusions:**

Water softeners provided no additional benefit to usual care in this study population. Small but statistically significant differences were found in some secondary outcomes as reported by parents, but it is likely that such improvements were the result of response bias, since participants were aware of their treatment allocation. A detailed report for this trial is also available at http://www.hta.ac.uk.

**Trial registration:**

Current Controlled Trials ISRCTN71423189

*Please see later in the article for the Editors' Summary*

## Abstract 2

In a randomized trial evaluating the effect of installation of ion-exchange water softeners in the households of children with eczema, the researchers found no evidence of improvement in eczema severity as compared to usual care in the study population.

## Editors' Summary

**Background:**

Eczema (sometimes referred to as atopic dermatitis) is a chronic, inflammatory skin condition that affects about 20% of school children in developed countries. Eczema is often associated with other conditions, such as asthma, hay-fever and food allergy and can cause intractable itching leading to thickened skin, bleeding, secondary infection, sleep loss, poor concentration, and psychological distress. Current topical treatments for eczema have side effects, for example, topical corticosteroids may cause skin thinning and the long term safety of topical tacrolimus and pimecrolimus has yet to be determined. Therefore, there is a lot of interest in exploring the benefits of non-pharmacological treatments that have no apparent side effects.

Water hardness (≥200 mg/l calcium carbonate) has become a recent focus of attention.

**Why Was This Study Done?:**

In addition to some epidemiological evidence linking increased water hardness with increased eczema prevalence, there have been widespread anecdotal reports of improvement in the skin of children with eczema when the family has moved from a hard to a soft water area. In addition, some patients report how their eczema symptoms have rapidly improved following the installation of a water softener. However, to date there have been no relevant published trials evaluating the potential benefit of water softeners for eczema. Given the lack of evidence, the high public interest in their potential benefit and the low risk of adverse effects, the researcher conducted a study to assess whether the installation of an ion-exchange water softener reduces the severity of eczema in children with moderate to severe eczema.

**What Did the Researchers Do and Find?:**

The researchers did a pilot study that showed that it was not possible to blind participants to their treatment allocation using real and “dummy” water softener units because the softened water produced more soap suds. So the researchers conducted an observer-blind randomised controlled trial in which they used trained research nurses to conduct an objective assessment of every participant's skin. The researchers recruited 336 children who all lived in hard water areas in England. Eligible children were aged 6 months to 16 years who had a diagnosis of eczema (in line with the UK working party's diagnostic criteria) and an eczema severity score of 10 or over. Participants were randomised to either installation of an ion-exchange water softener plus usual eczema care, or usual eczema care alone. Trained research nurses examined each child's skin at baseline and at 6, 12, and 16 weeks to record changes in eczema severity. The researchers also analysed any changes in symptoms over the study period such as, sleep loss and itchiness, the amount of topical corticosteroid/calcineurin inhibitors used, the Dermatitis Family Impact questionnaire and the health related Quality of Life (children's version).

Although both treatment groups improved in disease severity during the course of the trial, the researchers found no difference between the treatment groups in the main outcome—eczema severity. Similar finding were found for night movement (scratching) and the use of topical medications (creams/ointments applied to the skin), both of which were blinded to intervention status. Nevertheless, parents in the trial did report small health benefits, and just over 50% chose to buy the water softener at the end of the trial because of perceived improvements in the eczema and the wider benefits of water softeners. It is unclear how much of this effect can be explained by prior belief in the effectiveness of the water softeners for the treatment of eczema.

**What Do These Findings Mean?:**

The results of this study suggest that water softeners provide no additional clinical benefit to usual care in children with eczema so the use of ion-exchange water softeners for the treatment of moderate to severe eczema in children should not be recommended. However, it is up to each family to decide whether or not the wider benefits of installing a water softener in their home are sufficient to consider buying one.

**Additional Information:**

Please access these Web sites via the online version of this summary at http://dx.doi.org/10.1371/journal.pmed.1000395.

- The UK's NHS presents information on eczema for patients and families
- MedlinePlus gives information for patients, families, and caregivers on eczema and other similar conditions
- The National Eczema Society in the UK provides information and a helpline for eczema patients, families, and caregivers
- Medinfo provides information for eczema patients
- Wikipedia has more information about water softening (note that Wikipedia is a free online encyclopedia that anyone can edit; available in several languages)

## Introduction

Atopic eczema is a chronic, itchy, inflammatory skin condition that commonly involves the skin creases. It is associated with asthma, hay fever, food allergy, and atopy. The term atopic eczema is synonymous with atopic dermatitis. The World Allergy Organisation now suggests that the phenotype of atopic eczema should be called just eczema unless specific IgE antibodies are demonstrated [1], and we will use the term eczema throughout this report. Eczema is very common, affecting around 20% of school children in developed countries [2]. Eczema can cause intractable itching leading to thickened skin, bleeding, secondary infection, sleep loss, poor concentration, and psychological distress to the child and the entire family [3]. The cost of treating eczema is substantial, both for the health provider and for families [4],[5].

Epidemiological evidence linking increased water hardness with increased eczema prevalence was first demonstrated in an ecological study of 4,141 randomly selected primary school children living around Nottingham, UK [6]. The 1-year period prevalence of eczema was 17.3% in the hardest water category and 12.0% in the lowest (odds ratio 1.54, 95% confidence interval [CI] 1.19–1.99 after adjustment for confounders). Similar but smaller gradients have since been reported in Japan [7] and Spain [8]. It is possible that hard water could exacerbate eczema, because hard water results in more soap and detergent use, which can directly irritate the dry skin found in people with eczema. Soap also reacts with calcium in hard water to form small chalk particles which can irritate eczematous skin. Indirect effects such as enhanced allergen penetration due to a disruption in the skin barrier [9], and increased bacterial colonisation of the skin, are also plausible mechanisms of how hard water could worsen eczema symptoms [10].

Current pharmacological treatments for eczema have their limitations; topical corticosteroids may cause skin thinning [11], and the long-term safety of topical tacrolimus and pimecrolimus is still uncertain [11]. Given such concerns about pharmacological treatments, it is not surprising that interest in a nonpharmacological treatment that has no apparent side effects is high. There have been widespread anecdotal reports of improvement in the skin of people with eczema when moving from a hard- to a soft-water area. Anecdotal reports from patients also report rapid improvement in the symptoms of eczema following installation of a water softener. A previous systematic review of eczema treatments failed to identify any relevant trials evaluating the potential benefit of water softeners for eczema [12]. In view of the limited evidence for water softeners in eczema, the high public interest in their potential benefit, and low risk of adverse effects, the UK National Institute for Health Research Health Technology Assessment programme prioritised and commissioned the Softened Water Eczema Trial (SWET).

The SWET had two main objectives: (1) to assess whether the installation of an ion-exchange water softener reduces the severity of eczema in children with moderate to severe eczema, and if so, (2) to establish the likely cost and cost-effectiveness of the intervention.

## Methods

Details of the study protocol have been reported previously [13]. The study was approved by North West Research Ethics Committee (Ref 06/MRE08/77) and written informed consent was provided by the parent/caregiver of participating children (with signed assent from older children as appropriate). Copies of the trial protocol and CONSORT statement are available as supporting information (Text S1 and Text S2, respectively).

### Study Design

The SWET trial was an observer-blind, parallel-group randomised trial of 12 weeks duration, followed by a 4-week observational period. Participants were randomised to receive either immediate installation of an ion-exchange water softener plus their normal eczema care (Group A) or normal eczema care alone (Group B). Only one child per household was randomised into the study. The primary outcome was assessed at 12 weeks, after which time the water softeners were removed for participants in Group A, or installed for a period of 4 weeks for those in Group B. The observational period between weeks 12 and 16 was included as initial pilot work suggested that participants valued the opportunity to try a “real” softener for themselves, and because it provided an opportunity to look at speed of onset of benefit and duration of treatment effects.

The trial used an observer-blind design, as a previous pilot trial involving real and “dummy” units [14] suggested that it was not possible to blind participants to their treatment allocation because the softened water produced more suds. When a double-blind design is not possible, it is essential to ensure that the outcome assessment is free of observer bias [15]. We achieved this by using trained research nurses to conduct an objective assessment of the child's skin at baseline, 4, 12, and 16 weeks.

### Protocol

A full copy of the final trial protocol and the analysis plan are available at http://www.swet-trial.co.uk. Changes to the protocol following ethics committee approval in January 2007 included minor amendments to trial documents, the inclusion of amounts of topical medications as an additional secondary outcome measure, and an end of trial follow-up questionnaire. One of the secondary outcomes (patient-assessed global improvement in eczema) was replaced with broad categories as defined by the SASSAD score (the proportion of children who had a reasonable (≤20%), good (>20% and ≤50%) or excellent (>50%) improvement in SASSAD score), as this was felt to be more appropriate in a single-blind study. All amendments were implemented before breaking of the treatment allocation code and before finalising the analysis plan.

### Recruitment

Recruitment took place between May 2007 and June 2009, in eight UK centres (Nottingham, Cambridge, London [2 centres], Isle of Wight, Portsmouth, Lincoln, and Leicester). Participants were identified through secondary care referral centres, primary care, or in response to publicity. For those living in rented accommodation, approval to install the unit was obtained from the landlord of the property (including both private and Council tenants). All lived in hard-water areas (≥200 mg/l calcium carbonate) and had a home suitable for straightforward installation of a water softener.

Three hundred thirty-six children aged 6 months to 16 years were enrolled in the trial. All had a diagnosis of eczema according to the UK working party's diagnostic criteria [16] and a minimum eczema severity score of ten points using the Six Area Six Sign Atopic Dermatitis severity score (SASSAD) [17]. Children with a SASSAD score of less than ten points were excluded to avoid possible floor effects in measuring treatment response. Children were also excluded if they planned to be away from the home for >21 days during the 12-week study period (to ensure adequate exposure to the intervention), if they had taken systemic medication (e.g., cyclosporin or UV light therapy) in the last 3 months or oral steroids in the last 4 weeks, if they had started a new treatment regimen in the last 4 weeks, or if they already had a water treatment device installed in the home.

### Interventions

Ion-exchange water softeners plus usual eczema care were compared with usual eczema care alone. Ion-exchange water softeners use a synthetic polystyrene resin to remove calcium and magnesium ions from household water, replacing them with sodium ions, thus eliminating the hardness. The resin becomes depleted of sodium and is recharged using sodium chloride (common salt). To avoid favouring any one company, a generic unit was produced for the trial. The units met all necessary regulatory standards and were installed by trained water engineers according to British Water's code of practice [18]. Water samples were tested once a week to check that the units were working correctly. Any samples with a reading of >20 mg/l calcium carbonate were referred to the engineer for investigation.

For those allocated to Group A, a water softener unit was installed in the child's main residence as soon as possible after the baseline visit. All water entering the home was softened, with the exception of a drinking water tap at the side of the kitchen sink (unless this was refused by the participant or was technically too difficult to install). Participants were asked to bathe and wash their clothes in the usual way. A written booklet provided at the time of installation of the softeners gave general advice about use of the water softener. This included instructions to (1) check the salt regularly, (2) send water samples for analysis on a weekly basis, and (3) to reduce soap usage by at least half, in line with general advice on the use of water softeners in the home [19].

Participants allocated to delayed installation (Group B), received an active unit after the primary outcome had been collected at 12 weeks.

Both groups received a support telephone call from the coordinating centre at 8 weeks, and all participants continued with their usual eczema care for the duration of the trial. Usual care was defined as any treatment that the child was currently using to control their eczema (e.g., topical corticosteroids, emollients). Participants were discouraged from starting new treatments during the period of the trial. Any patients who started new treatments were defined as protocol violators and excluded from the per-protocol analysis.

### Outcomes

Primary outcome was the difference between Groups A and B in mean change in disease severity at 12 weeks compared with baseline, as measured using SASSAD [17]. This was chosen as the primary outcome because SASSAD has been used extensively in other clinical trials of eczema [20],[21] and because we had personal experience of using the scale in a clinical trial setting. It is easy and quick to complete, does not require an assessment of surface area involvement (which is extremely difficult to do reliably in patients with eczema) [22], and most importantly it is entirely performed by the observer, making it a good objective outcome measure for this observer-blind trial. Research nurses were trained in the use of the scale by either a dermatologist or a dermatology nurse consultant. Training was deemed complete when scores were <10% of each other. As far as possible the same nurse conducted baseline and follow-up assessments for individual participants. A reduction in the SASSAD score represents an improvement in eczema severity.

Secondary outcomes were differences in the following measures.

1. Proportion of time spent moving during the night. This is an objective surrogate for sleep loss and itchiness, which are two of the defining features of eczema [23],[24]. Nighttime movement was captured using wrist accelerometers (Actiwatch Mini, supplied by CamNtech Ltd, Cambridge, UK), and measured at baseline and at 12 weeks.
2. Proportion of children who had the same or a worse outcome (≤0%) or had a small (>0% and ≤20%), good (>20% and ≤50%), or excellent (>50%) improvement in SASSAD score.
3. Amount of topical corticosteroid or calcineurin inhibitors used.
4. Patient Oriented Eczema Measure [25].
5. Number of totally controlled week(s) (TCW) and well-controlled week(s) (WCW) [26].
6. Mean change in the Dermatitis Family Impact (DFI) questionnaire [27].
7. Mean change in health related quality of life (children's version of the EQ-5D for children aged 7 years and over, or the proxy version of the EQ-5D for children aged 3 to 6 years) [28].

In addition, a subgroup analysis was planned for participants with at least one of the two most common mutations of the gene encoding filaggrin (loss-of-function mutations R501X and 2282del4). Filaggrin is a protein whose deficiency might predispose to impaired skin barrier function and enhanced benefit from water softening [9].

All outcomes were collected during clinic assessments with the research nurse at baseline, 4, 12 and 16 weeks, or through daily diaries.

As the intervention involved the use of a commonly available household technology with no known side-effect, adverse events were not anticipated nor collected during the trial.

### Sample Size

Sample size estimates were based on published data relating to the use of SASSAD in patients recruited in secondary care [17],[21],[29]. Using an unpaired *t*-test with equal variance, a sample size of 310 children provided 90% power, with a significance level of 5% (assuming an attrition rate of 15%). This was based on a minimum clinically relevant difference between the groups of 20% in the change in SASSAD score, assuming a mean baseline score of 20 and no improvement in the usual-care arm. The standard deviation for the change in SASSAD score was assumed to be 10 [29].

For the planned subgroup analysis of children with at least one mutation in the filaggrin gene, a total of 90 children with the mutation was sufficient to detect a 30% difference between the treatment groups in the primary outcome, with 80% power, 5% significance, and a standard deviation of 10.

### Randomisation and Blinding

Participants were randomised using a web-based randomisation tool and were allocated on a 1∶1 basis according to a computer-generated code, using random permuted blocks of randomly varying size. The programme was created by the Nottingham Clinical Trials Unit (CTU), and held on a secure server. Randomisation was stratified by disease severity (baseline SASSAD ≤20, or SASSAD score >20) and recruiting centre. Access to the sequence was confined to the CTU Data Manager. The allocation group was indicated to the trial manager only after baseline data had been irrevocably entered into the randomisation programme. The sequence of treatment allocations was concealed until all interventions had been assigned and recruitment, data collection, and analysis were complete.

The research nurses were blinded to treatment allocation throughout the trial and the statistician analysed the results based on treatment code, using an analysis plan that was finalised before the coded allocation sequence was revealed. The only trial personnel in direct contact with participants were the research nurses and water engineers. The trial manager and study support staff at the coordinating centre in Nottingham had telephone contact with parents of participants. Trial participants continued to see their normal health care professionals for their ongoing eczema care.

Participants were discouraged from discussing their treatment allocation with the research nurse, and the importance of maintaining “blinding” was highlighted in the participant information sheets.

### Statistical Methods

Analyses were performed by CO (author) in Stata 10.1 [30]. Results were analysed based on treatment code, using an analysis plan that had been finalised prior to locking the database. Reported p-values are two-sided, with a significance level of 5%. The primary outcome was analysed as intent-to-treat (ITT) using the randomised treatment allocation rather than actual treatment received. Only participants with complete data were included in the ITT analysis, as the analysis plan specified that imputation of missing values was not required if less than 5% of data were missing. Baseline characteristics were summarised and an adjusted analysis was conducted where major imbalances existed. An additional per-protocol analysis was performed excluding protocol violators; these were determined before treatment allocation was decoded (for further details of protocol violators please see full trial report) [14].

The average percentage of the night spent moving was calculated by taking the average of the first 3 nights of usable data at baseline and the last 3 nights of usable data at week 12, as these days were closest to the nurses' assessments of eczema severity. Data were collected on the proportion of the night spent moving, regardless of sleep status (awake/asleep). Usable data were defined as values between 5% and 95% of the night spent moving to exclude outliers.

The total amount of medication used during the 12-week study period was measured by weighing the medication at each visit.

The number of TCW and WCW were compared. A TCW was defined as a week with 0 days with an eczema bother score >4 and 0 days in which “stepping up” of treatment was required. Stepping up of treatment was treatment over and above that defined as “normal” for an individual participant in the daily symptom diaries. Bother scores were assessed on a scale of 0 to 10 in answer to the following question: *How much bother has your child's eczema been today?* A WCW was defined as a week with ≤2 days with an eczema bother score >4 and ≤2 days where “stepping up” was required.

All other outcomes were scored according to the guidelines for the scale and compared the mean change from baseline to week 12. Continuous data were analysed using a *t*-test and categorical data were analysed using a Chi-squared test for trend.

## Results

A total of 336 participants were enrolled in the trial. Of those allocated to Group A, the water softeners were installed an average of 12 days after randomisation into the trial (SD 5.5). The average duration of installation was 74 days (SD 7.6). Twenty-one hardness alerts (sample readings of >20 mg/l calcium carbonate) were received during the 12-week trial period. These were resolved within 8 days on average (SD 4.5).

**Figure 1:**
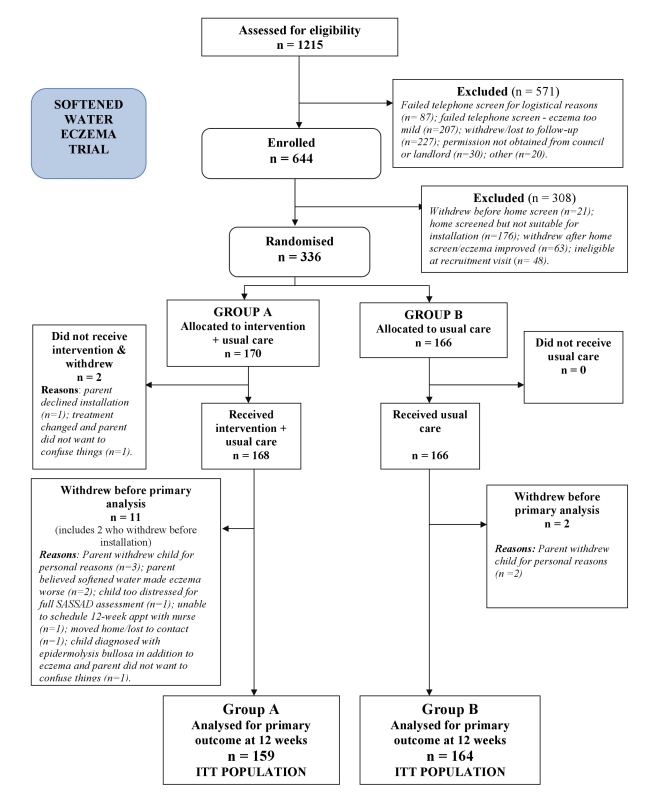
CONSORT flowchart.

The ITT population included 159 participants in Group A (water softener+usual care) and 164 in Group B (usual care). One participant was excluded because of incomplete data at baseline, and 12 participants withdrew from the trial before the primary endpoint at week 12 (Figure 1).

We found no difference between the groups in the primary outcome of disease severity. The mean change in the SASSAD score at 12 weeks compared to baseline was −5.0 (a 20% improvement) in Group A and −5.7 (a 22% improvement) in Group B. The mean change in disease severity between the two groups at 12 weeks was 0.66 (95% CI −1.37 to 2.69; *p* = 0.53) in favour of Group B. An additional analysis adjusting for stratification variables (baseline SASSAD and centre) was performed, but this did not alter the conclusion. The difference between the two groups was reduced to 0.34 (95% CI −1.65 to 2.33, *p* = 0.74) in favour of Group B.

The groups were broadly balanced at baseline in both clinical and demographic characteristics (Table 1). However, as a result of the slight imbalance between the groups in age, previous treatment history, and use of biological washing powder, a generalised linear model was used to adjust for these baseline differences. This analysis gave similar results to the univariate *t*-test analysis. The difference between the two groups was 0.54 (95% CI −1.54 to 2.62, *p* = 0.61) in favour of Group B. Additional sensitivity analyses excluding cases where the nurse became unblinded (*n* = 24), where a different nurse was required to perform the follow-up SASSAD assessment due to maternity leave (*n* = 14), and excluding outliers (change scores outside the range of ±3 SD) (*n* = 3), supported the primary ITT analysis.

**Table 1:** Baseline characteristics of study population.

Characteristics	Group A: Water Softener+Usual Care	Group B: Usual Care
***N* enrolled**	**170**	**166**
***N* in ITT population**	**159**	**164**
**Age**		
Mean age (SD)	5.8 (4.2)	5.1 (4.0)
**Sex, *N* (%)**		
Male	89 (56)	96 (59)
Female	70 (44)	68 (41)
**Ethnicity, *N* (%)**		
White	124 (78)	125 (76)
Non-white	34 (21)	38 (23)
Not stated/unknown	1 (1)	1 (1)
**Previous treatment history, *N* (%)** a		
High-strength corticosteroids/calcineurin inhibitors	91 (57)	80 (49)
Low-strength corticosteroids/calcineurin inhibitors	57 (36)	73 (45)
None	11 (7)	11 (7)
**Filaggrin status, *N* (%)**		
Presence of mutation	45 (28)	47 (29)
Absence of mutation	103 (65)	109 (66)
Unknown	11 (7)	8(5)
**Food allergy, *N* (%)** b		
No	97(63)	102 (64)
Yes	58 (37)	58 (36)
**Baseline SASSAD, *N* (%)** c		
Mean (SD)	24.6 (12.7)	25.9 (13.8)
Median (IQR)	21 (15–32)	22.5 (15.5–33.5)
10–19	72 (45)	68 (41)
>20	87 (55)	96 (59)
**Water hardness (mg/l** **calcium carbonate)**		
Mean (SD)	309 (50)	310 (58)
Median (IQR)	308 (274–342)	300 (270–340)
**Washing powder, *N* (%)** d		
Biological	20 (13)	12(7)
**Fabric softener, *N* (%)** e		
Yes	69 (44)	81 (49)
**Bathing freq at home, times per week** f		
Median (IQR)	5 (3–7)	4 (3–7)
**Bathing frequency away from home, times per week** g		
Median (IQR)	0 (0–1)	0 (0–0)
**Swimming frequency, *N* (%)** h		
Never	56 (35)	66 (40)
Less than once a month	53 (34)	52 (32)
More than once a month	49 (31)	46 (28)

a High-strength medication consists of those using potent or very potent steroids, or mild or moderate calcineurin inhibitors. Low-strength medication consists of those using mild or moderate steroids only.

b There were eight missing values for the food allergy variable.

c There was one missing value for SASSAD at baseline as the patient was randomised on the strength of a partial SASSAD score that excluded the child's legs.

d There were four missing values for the washing powder variable.

e There were three missing values for the fabric softener variable.

f There was one missing value for the bathing at home frequency variable.

g There were 12 missing values for the bathing away from home variable.

h There was one missing value for the swimming frequency variable.

IQR, interquartile range.

The additional per-protocol analysis excluding protocol violators also supported the primary ITT analysis (Table 2).

**Table 2:** Objective outcome measures (primary and secondary).

Measures	Group A: Water Softener+Usual Care	Group B: Usual Care	Difference and 95% CI (A–B)	*p*-Value
**Change in SASSAD score from baseline to week 12: Primary ITT analysis**				
*N* a	**159**	**164**		
Week 0, mean (SD)	24.6 (12.7)	25.9 (13.8)		
Week 12, mean (SD)	19.6 (12.8)	20.2 (13.8)		
Change, mean (SD)	−5.0 (8.8)	−5.7 (9.8)	0.66 (−1.37 to 2.69)	0.53
**Change in SASSAD score from baseline to week 12: Per protocol analysis**				
*N* b	**99**	**115**		
Week 0, mean (SD)	25.3 (13.7)	26.3 (14.5)		
Week 12, mean (SD)	20.8 (13.6)	20.0 (13.4)		
Change, mean (SD)	−4.5 (9.3)	−6.3 (9.9)	1.87 (−0.73 to 4.47)	0.16
**Change in SASSAD score from baseline to week 12 in participants who had at least one mutation on the filaggrin gene**				
*N* c	**45**	**47**		
Week 0, mean (SD)	27.2 (13.4)	26.7 (13.4)		
Week 12, mean (SD)	22.0 (13.4)	20.4 (13.9)		
Change, mean (SD)	−5.2 (9.5)	−6.3 (6.8)	1.05 (−2.36 to 4.47)	0.54
**Change in the percentage of the night spent moving**				
*N* d	**114**	**121**		
Week 0, mean (SD)	21.2 (7.7)	22.4 (9.7)		
Week 12, mean (SD)	24.7 (15.9)	26.5 (17.9)		
Change, mean (SD)	3.5 (14.5)	4.1 (16.8)	−0.64 (−4.68 to 3.40)	0.76
**Total amount (in grams) of all medication used between baseline and week12**				
*N* e	**160**	**153**		
Total medication used, mean (SD)	58.4 (96.8)	67.3 (97.3)	−8.90 (−30.50 to 12.70)	0.42

a Based on participants with evaluable data at baseline and week 12.

b Excluding participants deemed to be protocol violators by the Protocol Violators Group.

c Based on participants who had at least one mutation and data at baseline and week 12.

d Based on participants with at least 3 nights of evaluable data at baseline and week12.

e Based on participants with available data at week 12.

Overall, there were no statistically significant differences between the groups for any of the objective secondary outcomes. These were the grouped eczema severity scores, the time spent moving during the night, and use of topical medication (Tables 2 and 3). Small but statistically significant differences in favour of the intervention were observed in three of the four unblinded secondary outcomes that were recorded by the participants or their carers (Patient Oriented Eczema Measure; number of well- and totally controlled weeks; and DFI score) (Table 4).

**Table 3:** Categories of improvement in eczema severity (SASSAD) scores.

Level of Improvement	Group A: Water Softener+Usual Care	Group B: Usual Care	Total	*p*-Value
*N* randomised	170	166	336	
*N* a	159	164	323	
Same or worse (≤0%)	39 (25%)	42 (26%)	81 (25%)	
Small (>0% and ≤20%)	37 (23%)	30 (18%)	67 (21%)	
Good (>20% and ≤50%)	53 (33%)	56 (34%)	109 (34%)	
Excellent (>50%)	30 (19%)	36 (22%)	66 (20%)	0.62

a Number of participants with evaluable data at both week 0 and week 12.

**Table 4:** Un-blinded secondary outcome measures.

Measures	Group A: Water Softener+Usual Care	Group B: Usual Care	Difference and 95% CI (A–B)	*p*-Value
**Change in Patient Oriented Eczema score from baseline to week 12**				
*N* a	161	162		
Week 0, mean (SD)	16.8 (6.0)	16.6 (5.6)		
Week 12, mean (SD)	11.1 (7.1)	13.0 (6.7		
Change, mean (SD)	−5.7 (7.2)	−3.6 (6.7)	−2.03 (−3.55 to −0.51)	0.009
**Difference in the number of WCW** b				
*N* a	138	129		
WCW, mean (SD)	8.3 (3.8)	7.3 (4.1)	0.99 (0.04 to 1.95)	0.04
**Difference in the number of TCW** c				
*N* a	137	128		
TCW, mean (SD)	2.9 (3.5)	1.7 (2.8)	1.19 (0.43 to 1.95)	0.002
**Change in DFI score from baseline to week 12**				
*N* a	151	158		
Week 0, mean (SD)	10.0 (6.8)	11.2 (7.3)		
Week 12, mean (SD)	6.8 (6.0)	9.3 (7.1)		
Change, mean (SD)	−3.2 (6.2)	−1.8 (5.4)	−1.33 (−2.63 to −0.03)	0.05
**Change in EQ-5D score from baseline to week 12**				
*N* a	112	112		
Week 0, mean (SD)	0.690 (0.298)	0.693 (0.274)		
Week 12^d^, mean (SD)	0.810 (0.236)	0.759 (0.245)		
Change, mean (SD)	0.119 (0.269)	0.066 (0.250)	0.054 (−0.015 to 0.122)	0.124

a Number of participants with data at both baseline and week12.

b WCW = 2 days or less with an eczema bother score >4 and 2 days or less where stepping up of treatment was needed.

c TCW = 0 days with an eczema bother score >4 and 0 days where stepping up of treatment was needed.

d Increase in score represents an improvement in health-related quality of life.

Saliva samples were screened for the two most common mutations in the filaggrin gene, R501X and 2282del4. Of the 314 participants with test results, 94 (30%) had at least one mutation in the filaggrin gene.

The planned subgroup analysis including children with complete SASSAD data and at least one mutation of the filaggrin gene (*n* = 92) supported the primary analysis and showed no additional benefit for participants with filaggrin gene mutations (Table 2).

Adverse events were not formally collected as the trial involved the use of a commonly available domestic water softening unit, with provision for mains drinking water while the water softening unit was installed. Nevertheless, the parents of two participants believed their child's eczema had worsened as a direct result of installation of the water softener and asked to have the unit removed. Parents of a third participant expressed concern that the water softener appeared to be making their child's eczema worse, but continued to take part in the trial.

**Figure 2:**
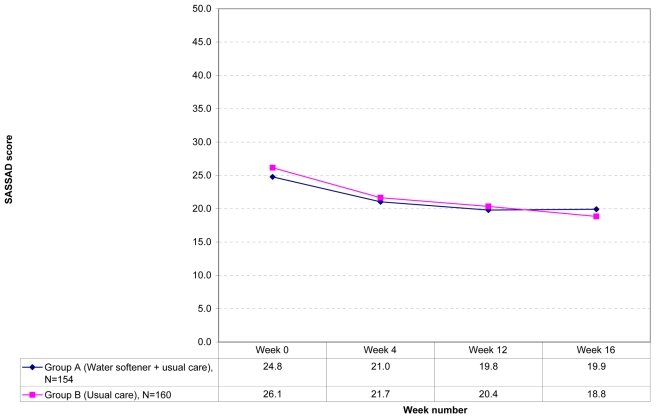
SASSAD scores during observational period (weeks 0 to 16).

Results of the cost-effectiveness analyses are available in the full trial write-up [14]. It was not appropriate to conduct analyses looking at possible duration of benefit and speed of onset of benefit in the final observational part of the study as there was no primary treatment effect. Nevertheless, the SASSAD scores collected between weeks 12 and 16 are shown for interest (when the softeners had been turned off for Group A and installed for Group B; Figure 2).

## Discussion

### Main Findings

This study found no benefit of using an ion-exchange water softener in addition to usual care in children with eczema. There were no clinically important differences between the treatment groups for any of the blinded outcomes. Furthermore, the 95% CIs around the primary efficacy estimates were narrow. An improvement of 1.37 points in favour of water softeners (the lower 95% CI), to 2.69 points in favour of usual care (the upper 95% CI) makes it unlikely that a clinically useful benefit has been excluded by chance. In order to understand the clinical relevance of these results it is helpful to consider the proportion of participants who showed good or excellent improvement during the period of the trial (52% in the water softeners group and 56% in the usual care group).

Performing a per-protocol analysis based on those with maximum exposure to the water softener, and excluding those who had changed their usual eczema treatments during the trial, did not change the overall interpretation of these results.

It is possible that water softeners could prove beneficial in the absence of a change in disease severity if the softeners resulted in a steroid-sparing effect. However, measurement of the amount of topical steroid or calcineurin inhibitors applied during the trial showed that both groups used roughly equivalent amounts of topical therapy throughout the 12-week study period.

Of the four unblinded secondary outcomes, all except EQ-5D showed small but statistically significant differences in favour of the water softener group. However, the magnitude of improvement seen in these outcomes was small and unlikely to be clinically significant. It is most likely that these differences were a result of response bias.

Of the children involved in the study, just under 30% had at least one filaggrin mutation, but these children showed no additional benefit compared to children without the mutation.

### Limitations

This was an adequately powered randomised trial, with high follow-up rates, that placed special emphasis on objective outcome measures to minimise response bias. Previous pilot work demonstrated the need for an objective outcome, as blinding participants with a sham unit was only partly successful (due to the different feel of softened water and the amount of suds generated) [14]. It is possible that our emphasis on objective outcomes meant that some important potential benefits were not captured in the primary analysis. Other factors, such as improvements in quality of life, or a reduction in symptoms (such as perception of skin dryness), may be important drivers in determining whether or not parents choose to buy a water softener. Indeed, many parents in the trial reported small health benefits, and just over 50% chose to buy the water softener at the end of the trial. The reasons participants gave for purchasing the units included perceived improvements in the eczema (66%); wider benefits of the softeners (27%); or both reasons (7%).

It is also possible that treatment effects were masked by the usual eczema care, but given the generally low use of topical corticosteroids and calcineurin inhibitors in both groups, this is unlikely to be the case.

This trial was of relatively short duration, and it is possible that the trial was insufficient to capture any treatment effect. However, both treatment groups improved in disease severity during the trial, and there was no hint that the intervention group was starting to show more improvement than the control group towards the end of the 12-week period. Anecdotal reports from patients returning from holidays claim benefits within 1–2 weeks. This led us to anticipate that if a treatment response existed, it was likely to occur more quickly than 12 weeks.

The continued use of soap and soap products during the trial may have limited the observed benefits if families were using too much soap in conjunction with the water softener. However, this pragmatic study aimed to capture the effects of water softeners according to standard advice. Evidence of how much soap was actually used was not collected, as we did not want to change participant's behaviour by intensive monitoring.

### Generalisability

We believe that this trial has good external validity, because participants were recruited from eight UK centres and included families of diverse socioeconomic backgrounds. Every effort was made to include participants who lived in rented accommodation as well as home owners. The results are applicable only to children with moderate to severe eczema, and it is possible that water softening is beneficial for milder forms of eczema, or in adults with other eczema types.

One possible reason for the discrepancy between our null trial findings and those of previous observational studies may be that water hardness has an effect on the primary prevention of eczema, rather than on the treatment of established eczema. The current study did not address the issue of prevention of new cases of eczema, which could be investigated by means of a further RCT including families at risk of eczema. An alternative explanation could be that the children in the observational studies ingested the water. In other words, it is possible that ingestion of hard water or a component to water that is related to water hardness actually induces skin inflammation directly or indirectly through inflammatory gene interactions, although we are not aware of any such potential mechanisms from the literature to date.

### Interpretation

The results of this study are clear, and as a result we cannot recommend the use of ion-exchange water softeners for the treatment of moderate to severe eczema in children. Whether or not the wider benefits of installing a water softener in the home are sufficient to justify the purchase of a softener is something for individual householders to consider on a case-by-case basis.

## Supporting Information

Text S1 Protocol and analysis plan.
(0.42 MB PDF)

Text S2 CONSORT checklist.
(0.22 MB DOC)

## Abbreviations

DFI: Dermatitis Family Impact
EQ-5D: Health-Related Quality of Life
ITT: intent-to-treat
SASSAD: Six Area Six Sign Atopic Dermatitis Score
TCW: totally controlled week(s)
WCW: well-controlled week(s)
