# Supplementary material for: A Randomised Controlled Trial of Ion-Exchange Water Softeners for the Treatment of Eczema in Children
Source: PLoS Med. 2011 Feb 15;8(2):e1000395. doi: 10.1371/journal.pmed.1000395 (PMC3039684; doi:10.1371/journal.pmed.1000395)
Supplement: Text S1 — Protocol and analysis plan. (0.42 MB PDF) [file pmed.1000395.s001.pdf]

# Softened Water Eczema Trial (SWET)

## A multi-centre randomised controlled trial of ion-exchange water softeners for the treatment of eczema in children

## Protocol

## Version 6.0
## 12 October 2009

**ISRCTN number:** ISRCTN71423189
**MREC Ref: 06/MRE08/77**
**Address of lead R&D:**
University Hospitals Nottingham, Research and Development, E11 Currie Court, Nottingham NG7 2UH

**Funded by:** NHS Health Technology Assessment (HTA) Programme

**Sponsored by**: University of Nottingham

**Co-ordinating Centre**:
Centre of Evidence Based Dermatology, University of Nottingham,
King's Meadow Campus, Lenton Lane, Nottingham. NG7 2NR

# CONTENTS


1. KEY CONTACTS .... 4
1.1 Trial Steering Committee .... 4
1.2 Trial Management Group .... 4
1.3 Other contributors to the study .... 5
2. BACKGROUND .... 6
2.1 Existing research .... 6
2.2 Hypothesis .... 7
2.3 Benefit / risk assessment .... 7
3. STUDY DESIGN .... 7
4. OBJECTIVES .... 8
5. INTERVENTIONS .... 8
5.1 Treatments to be compared .... 8
5.2 Treatment adherence / loss to follow-up .... 9
5.3 Concomitant therapy .... 10
5.4 Rescue medication .... 10
5.5 Starting and stopping treatment .... 10
6. OUTCOME MEASURES .... 10
6.1 Primary outcomes .... 10
6.2 Secondary outcomes .... 10
6.3 Further exploratory analyses .... 11
7. ELIGIBILITY CRITERIA .... 12
7.1 Inclusion criteria .... 12
7.2 Exclusion criteria .... 12
8. SCREENING AND RECRUITMENT .... 13
8.1 Participants .... 13
8.2 Setting .... 13
8.3 Randomisation and blinding .... 13
8.4 Screening .... 14
9. DATA COLLECTION .... 15
9.1 Data Collection Methods .... 15
9.2 Accelerometers .... 16
9.3 Telephone support .... 16
9.4 Engineer's visits .... 18
10. STATISTICS .... 18
10.1 Statistical design .... 18
10.2 Sample Size estimate .... 19
10.3 Primary analyses .... 19
10.4 Secondary analyses .... 19
10.5 Tertiary analyses .... 20
11. COST-EFFECTIVENESS ANALYSIS .... 20
11.1 Objective: .... 20
12. ADDITIONAL GENETICS STUDY .... 21
12.1 The role of filaggrin gene mutations as a predictor of treatment response .... 21
12.2 Methodology .... 22
12.3 Written informed consent .... 22
12.4 Sample size .... 22
13. TRIAL ADMINISTRATION .... 23
13.1 Trial personnel .... 23
13.2 Roles & responsibilities .... 23
13.3 Conflicts of interest .... 24
13.4 Trial Organisation & administration .... 24

13.5 Trial Timetable and Milestones ........ 25
13.6 Unblinding of participants ........ 25
14. ETHICS ........ 25
14.1 Statement of confidentiality ........ 25
14.2 Data protection ........ 25
15. ADVERSE EVENT REPORTING ........ 26
16. TRIAL INSURANCE AND INDEMNITY ........ 26
16.1 Negligent Harm ........ 26
16.2 Research Liabilities ........ 26
16.3 Non negligent harm ........ 26
17. PUBLICATION POLICY ........ 26
18. TRIAL FINANCES ........ 27
APPENDIX 1: Recruiting centres ........ 32
APPENDIX 2: R&D departments ........ 33

## Abbreviations

| | |
|---|---|
| GCP | Good Clinical Practice |
| CI | Chief Investigator |
| COREC | Central Office for Research Ethics Committees |
| CRF | Case Report Form |
| CTSU | Clinical Trials Support Unit |
| DFI | Dermatitis Family Impact (questionnaire) |
| Eczema | Atopic Eczema |
| EQ-5D | EuroQol 5 dimension (Quality of Life instrument) |
| HTA | Health Technology Assessment |
| ITT | Intention to Treat |
| MREC | Multi-centre Research Ethics Committee |
| MHRA | Medicines & Healthcare Products Regulatory Agency |
| PI | Principal Investigator |
| POEM | Patient-Oriented Eczema Measure |
| SASSAD | Six Area Six Signs Atopic Dermatitis Score |
| SOP | Standard Operating Procedure |
| TCW | Totally Controlled Weeks |
| TMG | Trial Management Group |
| TSC | Trial Steering Committee |
| WCW | Well Controlled Weeks |

# 1. KEY CONTACTS

## 1.1 Trial Steering Committee

| Name & Address | Tel | E-mail |
|---|---|---|
| Dr David Paige (TSC Chair)<br>Consultant Dermatologist<br>The Royal London Hospital, London | 0207 377 7383 | David.paige@bartsandthelondon.nhs.uk |
| Prof Hywel Williams (Chief Investigator)<br>Consultant Dermatologist<br>Centre of Evidence Based Dermatology<br>University of Nottingham | 0115 823 1047 | Hywel.williams@nottingham.ac.uk |
| Dr Ian Pollock (Principal Investigator)<br>Consultant Paediatrician<br>Barnet & Chase Farm Hospital, London | 0208 375 1900 | Ian.pollock@bcf.nhs.uk |
| Mr David Potter (Consumer representative)<br>Research Biochemist (retired) | 01795 473302 | dandsap@talktalk.net |
| Prof Andrew Nunn (Statistician)<br>MRC Clinical Trials Unit, London | 0207 670 4703 | Andrew.nunn@ctu.mrc.ac.uk |
| Dr Nerys Roberts (TSC Deputy Chair)<br>Consultant Dermatologist<br>Chelsea & Westminster Hospital, London | 0208 746 5293 | n.roberts@doctors.net.uk |
| Dr Ian Pallett (Water Industry Representative)<br>British Water, London | 0207 957 4554 | Ian.pallett@britishwater.co.uk |
| Dr Karin Koller (Trial Manager)<br>Centre of Evidence Based Dermatology<br>University of Nottingham | 0115 846 8623 | Karin.koller@nottingham.ac.uk |

## 1.2 Trial Management Group

| Name & Address | Tel | E-mail |
|---|---|---|
| Prof Hywel Williams (Chief Investigator)<br>Consultant Dermatologist<br>Centre of Evidence Based Dermatology<br>University of Nottingham | 0115 826 1047 | Hywel.williams@nottingham.ac.uk |
| Dr Kim Thomas (Lead applicant)<br>Deputy Director, Centre of Evidence Based Dermatology, University of Nottingham | 0115 846 8632 | Kim.thomas@nottingham.ac.uk |
| Dr Sarah Meredith<br>Senior Clinical Epidemiologist<br>MRC Clinical Trials Unit, London | 0207 670 4700 | sarah.meredith@ctu.mrc.ac.uk |
| Professor Andrew Nunn<br>Associate Director, MRC Clinical Trials Unit, London | 0207 670 4703 | andrew.nunn@ctu.mrc.ac.uk |
| Dr Angela Crook<br>MRC Clinical Trials Unit, London | 0207 670 4703 | Angela.crook@ctu.mrc.ac.uk |
| Dr Caroline Gilbert<br>MRC Clinical Trials Unit, London | 0207 670 4703 | Caroline.gilbert@ctu.mrc.ac.uk |

| | | |
|---|---|---|
| Dr Ian Pollock (Principal Investigator)<br>Consultant Paediatrician<br>Barnet & Chase Farm Hospital, London | 0208 375 1900 | ian.pollock@bcf.nhs.uk |
| Dr Nigel Burrows (Principal Investigator)<br>Consultant Dermatologist<br>Addenbrookes Hospital, Cambridge | 01223 216 501 | nigel.burrows@addenbrookes.nhs.uk |
| Professor Tara Dean (Principal Investigator)<br>Reader in Epidemiology/Deputy Director of Asthma & Allergy Research Centre<br>St Mary's Hospital, Isle of Wight | 023 9284 4405 | tara.dean@port.ac.uk |
| Dr Robin Graham-Brown (Principal Investigator)<br>Consultant dermatologist/Hon Senior Lecturer<br>University Hospitals of Leicester NHS Trust | 0116 258 5384 | robin.grahambrown@uhl-tr.nhs.uk |
| Dr Mansoor Dilnawaz (Principal Investigator)<br>Consultant Dermatologist, Pilgrim Hospital, Boston<br>United Lincolnshire Hospitals NHS Trust | 01205 446436 | Mansoor.dilnawaz@ulh.nhs.uk |
| Dr Edel O'Toole (Principal Investigator)<br>Consultant Dermatologist, The Royal London Hospital | 020 7882 2483 | e.a.otoole@qmul.ac.uk |
| Dr Tracey Sach<br>Senior Lecturer in Health Economics<br>University of East Anglia | 01603 592022 | t.sach@uea.ac.uk |
| Grant Audemard (Water Softener Industry Rep)<br>Kinetico UK Ltd , Park Gate, Hampshire | 01489 566970 | gaudemard@kinetico.co.uk |
| Tony Frost (Water Softener Industry Rep)<br>Aqua Focus Ltd, Newport, Shropshire | 01952 691219 | Tonyfrost@aquafocus.co.uk |
| Dr Karin Koller (Trial Manager)<br>Centre of Evidence Based Dermatology<br>University of Nottingham | 0115 846 8623 | Karin.koller@nottingham.ac.uk |
| Jane Grundy (Research Nurse)<br>St Mary's Hospital, Isle of Wight | 01983 534178 | Jane.grundy@iow.nhs.uk |
| Rhiannon Medhurst (Research Nurse)<br>Barnet & Chase Farm Hospital, London | 0208 375 2398 | Rhiannon.medhurst@bcf.nhs.uk |
| Rosalind Simmonds (Research Nurse)<br>Addenbrooke's Hospital, Cambridge | 01223 216465 | rosalind.simmonds@addenbrookes.nhs.uk |
| Susan Davies-Jones (Research Nurse)<br>QMC and University of Nottingham | 07982 466230 | Sue.davies-jones@nottingham.ac.uk |
| Amanda Roper (MCRN-funded Research Nurse)<br>United Lincolnshire Hospital NHS Trust | 01522 573096 | Amanda.Roper@ulh.nhs.uk |
| Alison Allen (MCRN-funded Research Nurse)<br>Barnet & Chase Farm Hospital, London | 0207 3777000<br>xtn 3940 | alison.allen@bartsandthelondon.nhs.uk |
| John Kyle (Engineers main contact, mainland)<br>Kinetico UK Ltd , Park Gate, Hampshire | 01489 566973 | jkyle@kinetico.co.uk |
| John Bisset, Engineering Services Manager<br>Kinetico UK Ltd , Park Gate, Hampshire | 01489 566970 | jbisset@kinetico.co.uk |
| Robin Stevens (Engineers main contact Isle of Wight),<br>MG Heating, Newport, IOW | 01983 537331 | robin@mgheating.co.uk |

## 1.3 Other contributors to the study

| | | |
|---|---|---|
| Mr Paul Cartledge<br>Head of Research Grants & Contracts,<br>University of Nottingham | 0115 951 5679 | Paul.cartledge@nottingham.ac.uk |

| | | |
|---|---|---|
| Dr Alan Irvine,<br>Consultant Paediatric Dermatologist,<br>Our Lady's Hospital, Crumlin, Dublin | + 353 1 428 2532 | alan.irvine@olhsc.ie |
| Mr Par Khosa<br>Finance Department<br>University of Nottingham | 0115 951 5792 | Par.khosa@nottingham.ac.uk |
| Mr Stephen Lemon<br>Monitoring Manager<br>Health Technology Assessment , Southampton | 023 8059 5616 | S.P.Lemon@soton.ac.uk |
| Professor W.H.Irwin McLean<br>Professor of Human Genetics,<br>University of Dundee | 01382 425618 | w.h.i.mclean@dundee.ac.uk |
| Professor Mark Sculpher<br>Professor of Health Economics, University of York | 01904 321401 | Mjs23@york.ac.uk |
| Mr Daniel Simpkins<br>Data Manager, University of Nottingham CTSU | 0115 823 0508 | Daniel.simpkins@nottingham.ac.uk |

# 2. BACKGROUND

## 2.1 Existing research

The NHS Health Technology Assessment (HTA) systematic review of atopic eczema treatments included a chapter on the evidence base for non-pharmacological interventions[1]. This failed to identify any trials evaluating the use of water softeners for patients with atopic eczema. The only trials of possible relevance were an inconclusive trial looking at the benefits of salt baths, and another that examined the use of biological versus non-biological washing powders. This search was updated in 2006 and no new references were found.

There is epidemiological evidence linking increasing water hardness with increasing atopic eczema prevalence. This was first demonstrated by the current research team in an ecological study published in *The Lancet* of 4141 randomly selected primary school children in the Nottingham area [2]. The 1-year period prevalence of eczema was 17.3% in the hardest water category and 12.0% in the lowest (odds ratio of 1.54, 1.19-1.99 after adjustment for confounders). Similar results have recently been found in Japan [3].

If the above associations are true, a number of plausible mechanisms can be forwarded to suggest why hard water could exacerbate eczema. Perhaps the most likely explanation is increased soap usage in hard water areas; the deposits of which can cause skin irritation in eczema sufferers. A direct chemical irritant effect from calcium and magnesium salts is also possible, or an indirect effect of enhanced allergen penetration from skin barrier disruption.

### 2.2 Hypothesis

- That the installation of an ion-exchange water softener will help to relieve the symptoms of eczema in children with moderate to severe eczema.
- That the installation of an ion-exchange water softener will result in cost implications to both patients and the NHS.

### 2.3 Benefit / risk assessment

This is very low risk trial as the intervention is non invasive with no known clinical side effects. Participants simply receive softened water for bathing and washing of clothes. Drinking water will remain unchanged.

The water softening units will be installed by a qualified water engineer according to the Code of Practice produced by British Water.

The unit to be used is a generic version of a commercially available unit which has been encased in a generic outer box in order to prevent commercial advantage for any individual water softener supplier. Ion-exchange technology is well understood and widely used throughout the world.

Possible benefits to trial participants, in addition to improvement in eczema symptoms, include reduced scaling of water appliances, reduced soap / cleaning product consumption and reduced energy consumption.

## 3. STUDY DESIGN

This is a single-blind, parallel group randomised controlled trial of 12-week duration, followed by a 4-week cross-over period (Figure 1). The study will be analysed as a parallel group study, but the final 4 week period will include exploratory analyses to explore within person effects. Specifically, these exploratory analyses will provide further information on (i) the speed of onset of benefit for the delayed treatment group, and (ii) on how quickly benefits are lost once treatment is removed in the active treatment group. Three hundred and ten children with moderate to severe eczema will each be enrolled into the study for a period of 16 weeks. Participants will be enrolled over a period of 18-20 months, starting in Spring 2007. The end of study is defined as being the final assessment visit of the last participant into the trial.

*Figure 1: Study design*

| | STUDY PERIOD = 16 weeks | | |
|---|---|---|---|
| | **0 to 12 weeks** | **12 to 16 weeks** | |
| **Group A** | Usual eczema care + water softener installed (n = 155) | Unit removed | Option to purchase unit at reduced cost |
| **Group B** | Usual eczema care + delayed installation (n = 155) | Unit installed | |

# 4. OBJECTIVES

i. To assess whether the installation of an ion-exchange water softener improves eczema in children.
ii. If so, to establish the likely cost and cost-effectiveness of this intervention.

# 5. INTERVENTIONS

## 5.1 Treatments to be compared

Ion-exchange water softening units will be compared with usual care. Ion exchange water softening is a scientifically defined, understood and described process using a synthetic polystyrene resin in which primarily the divalent cations (positively charged), calcium and magnesium found in domestic water supplies, are replaced by the monovalent cation, sodium, from common salt. The water softener used in the study has two cylinders of resin which are used alternately. A control valve ensures that when the resin capacity of one cylinder is exhausted it automatically switches the water flow to the second cylinder and, using common salt, regenerates the first to be ready for use when the second is exhausted. Ion-exchange water softening units typically reduce the water hardness to practically zero[1].

All units will be installed in the child's principal residence and salt will be supplied for the duration of the trial. Standard procedure will be to soften all water in the home, and provide mains drinking water through an extra (faucet-style) tap installed at the side of the kitchen sink. Participants will be given the opportunity to opt out of having this

[1] By contrast, physical water conditioners affect the behaviour of calcium in water but they do not remove it. The process by which they have this effect is not fully understood scientifically and so their design is empirical. Whether or not Physical Water Conditioners have a beneficial effect on eczema is a question for future study once proof of principle has been tested using established ion-exchange technology.

separate, mains drinking water tap if they prefer.

Apart from having a unit installed in the home, participants will continue with their usual eczema treatments in the usual way and will be asked to bathe / wash their clothes according to their usual practice. The units will meet all necessary quality standards, and will be installed by a trained water engineer according to British Water's code of practice.

The water softeners to be used in this trial will be supplied and paid for by a consortium of representatives from the water treatment industry, co-ordinated through their Trade Association. The units will be encased in an unmarked box in order to prevent the possibility of commercial advantage to any particular company. Similarly, unmarked salt will be supplied for use during the trial.

Participants allocated to delayed installation will subsequently receive an active unit at week 12

During the pilot study, approval was sought from the Housing Departments of local Councils to install units in the homes of Council tenants. This was very successful and will be used again in the main trial in order to be as inclusive as possible with regards to trial participants. The relevant water companies will also be informed of the trial, and will be provided with details of the likely number of water softeners to be installed in each region.

### 5.2 Treatment adherence / loss to follow-up

Compliance with treatment does not represent a large problem for this trial as long as the participants are not absent from home for long periods of time. With the exception of a drinking water tap, the water is simply softened (or not) for the entire household. However, participants must remember to periodically replenish the salt (every few weeks) and it is possible that this may not be done. Evidence from the pilot study suggested that some families did use less salt than others.

In order to assess that the units are working correctly, water samples will be sent to the research team once a week. Any samples with a reading of > 20 mg/L calcium carbonate will be referred back to the engineer for investigation. Participants will also be reminded of the importance of replenishing the salt supply by telephone at 8 weeks. A weekly reminder will also be included in the child's symptom diary.

It is anticipated that loss to follow-up will be <15%. For the pilot study all of the children attended for their final appointment. Nevertheless, a previous 18-week study of treatments for children with atopic eczema run by the investigators resulted in a loss to follow-up of 15% [4] and we propose to adopt this as a more conservative estimate. At the end of the study all participants will be offered the chance to purchase the units at a reduced cost (£446.50 inclusive of VAT, installation and warranty; this is approximately half the full retail price).

### 5.3 Concomitant therapy

Participants will be allowed to use their usual eczema treatments as prescribed. However, children will be asked not to start any NEW treatments during the period of the study if medically possible. (*See also* exclusion criteria).

### 5.4 Rescue medication

Rescue medication will be defined by asking participants at the recruitment visit what they would do if they needed to "step-up" their treatment in response to a worsening of the eczema. The need to "step-up" treatment will then be recorded in the child's diary on a daily basis.

### 5.5 Starting and stopping treatment

Units will be installed in the participants' homes as soon as possible after being randomized to treatment (ideally within 10 working days).

If participants choose to withdraw from the study, any units that have been installed will be removed as soon as is practicably possible. Participants will be asked to complete an end of study questionnaire at this time and diaries will be collected.

If participants are away from the main residence for any reason, this information will be recorded in their treatment diaries. Absence from the home will be included in a predictors of response model and will be used as a measure of treatment adherence for the (secondary) per protocol analysis.

## 6. OUTCOME MEASURES

### 6.1 Primary outcomes

1. Difference between the active vs. standard treatment groups with regard to mean change in disease severity (Six Area Six Sign Atopic Dermatitis Score – SASSAD [5]) at 12 weeks compared to baseline. SASSAD is an objective severity scale that is completed by the research nurse during follow-up appointments. It does not involve input from the participant in any way.

### 6.2 Secondary outcomes

1. Difference between the groups in the proportion of time spent moving during the night[2]. Movement will be captured for periods of one week at week 1 and week 12, and will be measured using accelerometers (Actiwatch™). These units are worn by the child in the same way as a wrist watch

[2] This outcome has been included as an objective surrogate for sleep loss and itchiness (two of the defining features of eczema). Previous research has suggested that this is a suitable objective tool for assessing itch [6,7] and further pilot work is currently underway to assess its suitability for use within this trial (results available Dec 2006)

This outcome has been included as an objective surrogate for sleep loss and itchiness (two of the defining features of eczema). Previous research has suggested that this is a suitable objective tool for assessing itch [6,7] and further pilot work is currently underway to assess its suitability for use within this trial (results available in 2007).

2. Difference in proportion of children who report either good or excellent improvement in eczema severity at 12 weeks (using a 5-point Likert scale).

3. Difference in the amount of topical corticosteroid / calcineurin inhibitors used during the 12 week study period.

4. Difference in Patient Oriented Eczema Measure (POEM [8]) collected at baseline, weeks 4, 12 and 16. This scale is a well validated tool that has been developed to capture symptoms of importance to patients (rather than objective signs that are used in traditional severity scales, such as SASSAD).

5. Difference in the number of totally controlled weeks (TCW) and well controlled weeks (WCW) based on the number of days with eczema symptoms and the number of days that topical treatment is applied. This outcome is based on a recent systematic review conducted by the applicants looking at ways of assessing long-term control for chronic conditions such as atopic eczema, asthma and rheumatoid arthritis [9]. The terms TCW and WCW have been adopted for use by researchers in the field of asthma and appear to be a useful and intuitive means of capturing disease activity over time.

6. Difference in the mean change in the Dermatitis Family Impact (DFI) questionnaire at 12 weeks [10]. This scale was chosen as an appropriate quality of life scale for the study for two reasons:

   - *The intervention involves the entire household, so a quality of life scale appropriate to the family unit seems most appropriate.*

   - *It avoids the need to use two different age-specific dermatology quality of life scales (the Children's Dermatology Life Quality Index[11] and the Infants version of the same scale[12]).*

7. Mean change in health related Quality of Life at 12 weeks. This will be captured using a generic measure of health utility (the children's version of the EQ-5D for children aged 7 years and over, or the proxy version of the EQ-5D for children aged 3 to 6 years [13]).

### 6.3 Further exploratory analyses

In addition to the main outcomes listed above, further exploratory analyses are planned as follows

- Difference in mean change in disease severity (SASSAD) at 4 weeks compared to

baseline. This outcome is included in order to capture speed of onset of benefit.

- Further within person analyses will be conducted comparing outcomes collected during the final 4-week period (12 to 16 weeks), with those collected during the initial 4 weeks of the study (0 to 4 weeks). Data collected for the active treatment group will provide an indication of the likely carry-over effect of this intervention, which will be useful in planning the design of future trials in this area. Data collected for the delayed treatment group will inform the analysis regarding speed of onset of improvement.

- Predictors of response model – including baseline factors such as filaggrin status (see later section), baseline eczema severity, water hardness, swimming activity and time away from the home).

# 7. ELIGIBILITY CRITERIA

The intention is to keep entry criteria as broad as possible in order to improve the external validity of the trial and to boost recruitment.

## 7.1 Inclusion criteria

- Children aged 6 months to 16 years at baseline, with eczema as defined by the UK refinement of the Hanifin and Rajka diagnostic criteria [14].
- Eczema present at time of assessment (minimum SASSAD score of 10).
- Baseline water hardness of >200 mg/L of calcium carbonate.
- Home suitable for the installation of a water softening device (as assessed by water engineer)

Only one child will be enrolled per family. The choice as to which child becomes involved will be made by the parents and children involved, taking into account the inclusion criteria above.

## 7.2 Exclusion criteria

- Children who plan to be away from home for >21 days in total during the 16-week study period. This has been deemed necessary in order to ensure adequate exposure to the intervention. We will also aim to ensure children do not have a planned holiday in the 4 weeks prior to their 12 week assessment visit.
- Children who have taken systemic medication (e.g. Cyclosporin A, methotrexate) or UV light for their eczema within the last 3 months because of their long lasting effects.
- Children who have taken oral steroids within the last 4 weeks, or who, as a result of seeing a healthcare professional, have started a new treatment regimen for eczema within the last 4 weeks.
- Families who already have a water treatment device installed, including ion-exchange softeners, polyphosphate dosing units or physical conditioners.

# 8. SCREENING AND RECRUITMENT

## 8.1 Participants

Children aged 6 months to 16 years with atopic eczema will be enrolled into the study. A diagnosis of eczema will be standardised using the UK working party's diagnostic criteria for atopic eczema [14]. It is anticipated that participants will be recruited at a rate of 4-5 per centre per month and that recruitment will take place over 18 to 20 months.

## 8.2 Setting

Recruitment will take place in eight secondary care referral centres in the UK serving a variety of ethnic and social groups and including both urban and peri-urban dwellings. All have predominantly hard water (although water in the Nottingham area is mixed). Inclusion criteria assume baseline water hardness of >200 mg/L for entry into the trial. The eight recruiting centres will be i) Queen's Medical Centre, Nottingham; ii) Barnet & Chase Farm Hospital, London; iii) Addenbrookes Hospital, Cambridge; iv) The David Hide Asthma and Allergy Research Centre, St Mary's Hospital, Isle of Wight Healthcare Trust, Isle of Wight, v) University Hospital of Leicester NHS Trust, vi) St Mary;s Hospital, Portsmouth, vii) United Lincolnshire Hospitals NHS Trust and (viii) The Royal London Hospital. All centres hold designated paediatric clinics in which children with eczema are commonly seen.

Participants will be informed of the trial in various ways. In order to “kick start” recruitment during the initial phase, patients who have been referred to the recruiting centres over the previous 12-18 months will be sent letters and information sheets about the trial. On-going recruitment will also take place through outpatient clinics, although children who have recently seen a dermatologist (and have started on a new treatment regimen), will not be able to enter the trial for at least 4 weeks. In addition, R&D approval will be sought from Primary Care Trusts local to the seven recruiting centres, and letters and information sheets sent to patients under the dermatological care of their GP. In addition, primary schools will be informed of the trial and efforts will be made to advertise the study through direct advertising in the local media, and on relevant websites. Recruitment in Nottingham will be limited to those areas with a hard water supply based on postcode areas.

## 8.3 Randomisation and blinding

Participants will be entered into a web-based randomisation programme by the Trial Manager, or Research Nurse. This will randomise them to one of the 2 treatment arms based on a computer generated code, using random permuted blocks of randomly varying size. This will be created by the Nottingham Clinical Trials Support Unit (CTSU) in accordance with their standard operating procedure, and held on a secure server. The randomisation will be stratified by age, disease severity (baseline SASSAD ≤ 20, or SASSAD score >20) and recruiting centre. Access to the sequence will be confined to the CTSU Data Manager. Allocation to treatment arms will be in the ratio 1:1 and the Trial Manager will access the treatment allocation for each participant by means of a remote,

internet-based randomisation system developed and maintained by the Nottingham CTSU. The allocation group will be indicated to the Trial Manager only after baseline data have been irrevocably entered into the randomisation programme. The sequence of treatment allocations will be concealed until interventions have all been assigned and recruitment and data collection are complete.

The research nurses will be blinded to treatment allocation throughout the study period and the trial statistician will analyse the results based on treatment code, using an analysis plan finalised prior to revealing the coded allocation sequence. Only after the analysis is complete will the actual treatment arms corresponding to treatment codes be revealed. The only study personnel in direct contact with study participants will be the research nurses and water engineers. The trial manager and study support staff at the co-ordinating centre in Nottingham will have telephone contact with parents of participants. Trial participants will continue to see healthcare professionals for their usual eczema care.

Since participants will not be blinded to the study intervention, an objective primary outcome has been chosen in order to minimise response bias, The primary outcome of disease severity, measured using the Six Area Six Signs Atopic Dermatitis scale (SASSAD) will be assessed by the research nurses who are blind to treatment allocation. This scale is based purely on physical examination of the skin and does not require input from the participants themselves. Other secondary objective outcomes include nocturnal movement (measured using accelerometers) and the use of topical therapy.

Participants will be discouraged from discussing which treatment they have received with the research nurse. In order to reduce the opportunity for 'un-blinding', telephone contacts will be conducted by the (un-blinded) trial manager whenever possible and participants will be reminded not to mention their treatment allocation to the research nurse prior to their assessment visits. The importance of maintaining 'blinding' will also be highlighted in the participant information sheets.

Integrity of information bias will be assessed using clinical photographs of a target lesion. These images will be taken at each assessment visit and graded remotely by 2 independent dermatologists. These dermatologists will not be aware of the study design, or of the assessment visit at which the image was taken. Should the two dermatologists score an image very differently, a 3rd dermatologist will be asked to adjudicate. This method of photo assessment worked well in the pilot study.

### 8.4 Screening

Screening for the trial will be a 3-stage process. Those who express a willingness to take part in the trial will be approached as follows (NOTE: the order of the contacts may vary depending on the recruitment route):

1. By telephone or postal questionnaire – participants will be sent a Participant Information Leaflet along with a screening checklist. This will be followed up by telephone to assess eligibility. Participants will give either written or verbal agreement at this time for the study's water engineer to visit their home.

2. Home visit by the engineer – the home will be assessed for suitability of installation of a water softening device.

3. Appointment with the research nurse - assessment of eligibility criteria and recruitment into the trial. This appointment will be carried out in outpatients or in the patient's home.

Consent will be taken by the research nurse prior to conducting the recruitment assessment. Five copies of the consent forms will be generated for: i) the participants; ii) the medical notes; iii) the child's GP; iv) the Trial Master File and v) the local Site File.

# 9. DATA COLLECTION

## 9.1 Data Collection Methods (summarised in Table 1)

### *9.1.1.Face-face interviews with the research nurse*

These interviews will take place at baseline (recruitment visit), 4 weeks, 12 weeks and 16 weeks.

The visits will include the following:

i. check of eligibility criteria (recruitment visit only)
ii. baseline characteristics / demographics (recruitment visit only)
iii. examining the child for disease severity (using the Six Area, Six Sign Atopic Dermatitis score)
iv. eczema symptoms (POEM)
v. interviewing the family for quality of life – dermatitis family impact questionnaire and EQ-5D
vi. willingness to pay for an ion-exchange water softener (recruitment and by letter sent at 10 -12 weeks)
vii. digital image of nominated target lesion[3]
viii. weighing medications.

### *9.1.2. Treatment diaries*

Diaries will be used to capture:

i. the number of days when active topical treatment (topical corticosteroids, tacrolimus or pimecolimus) is applied
ii. whether or not treatment has needed to be "stepped up" – if so, in what way and for how many days ("stepping up" treatment will be defined in advance by the parents in consultation with the research nurse)
iii. daily global assessment of disease activity by child and parents
iv. nights away from home

---

[3] Target lesion nominated by research nurse in discussion with the child/parents.

v. health service resource use
vi. personal costs associated with the eczema
vii. whether or not the accelerometers have been worn (weeks 1 and 12).

Items i) and ii) above will be used to calculate the number of totally controlled weeks (TCW) and well controlled weeks (WCW) during the study. Diaries will also be used to remind participants to check the salt levels in the machine and to return a sample of the water for testing in the laboratory every week.

### 9.2 Accelerometers

Night-time movement will be measured each night for a period of 1 week at the beginning of the study (week 1) and at the end of the 12-week study (week 12). Data will be stored on the Actiwatch™ units and downloaded onto a laptop computer at the subsequent assessment visits. Recordings will take place every night between the hours of 10pm and 6am, as used by other investigators[6].

### 9.3 Telephone support

Participants will be contacted by the trial manager at 8 weeks in order to provide advice and support about the study.

### 9.4 End of Trial Follow-up Questionnaire

Parents of participants will be sent an end of trial follow-up questionnaire once all participants have completed the study. This will seek information about current eczema status, and whether or not they have a functioning water softener.

Table 1: Summary of schedule of assessment visits

| | **BASELINE**<br>**= Day ZERO** | **Group A**<br>**installation** | **"4 week" Appointment** | **"8 week"**<br>**Reminder** | **"12 week"**<br>**Appointment** | **Group A Un-installation Group B Installation** | **"16 week"**<br>**Appointment** |
|---|---|---|---|---|---|---|---|
| Water Engineer Home screening visit | Research Nurse Recruitment Visit clinic appointment | Water Engineer<br>At 0–2 weeks | Research Nurse Clinic appointment At 4–6 weeks | Trial Manager<br>At 8 to 9 weeks | Research Nurse Clinic appointment At 12–14 weeks | Water Engineer<br>At 12–14 weeks | Research Nurse Water Engineer At 16 to 18 weeks |
| ◊ Home visit to assess suitability of residence for installation of softener<br>◊ Estimate salt requirements and assess availability of storage for salt supply<br>◊ Test water hardness | ◊ Baseline characteristics<br>◊ Eligibility criteria checked<br>◊ SASSAD<br>◊ POEM<br>◊ Utility measures (DFI and EQ-5D)<br>◊ Weigh medications<br>◊ WTP questionnaire<br>◊ Digital photo of index site<br>◊ Saliva sample<br>◊ Explanation and issue of Diary 1<br>◊ Explanation of accelerometer (Actiwatch™)<br>◊ Issue Water Softener Information sheet<br>◊ Randomisation | ◊ Install unit (Group A) within 10 working days of DAY ZERO (date of recruitment visit)<br>◊ Advice given to reduce soap consumption etc.<br>◊ Issue water sampling containers + prepaid envelopes | ◊ SASSAD<br>◊ POEM<br>◊ Digital photo of index site<br>◊ Saliva sample (if not obtained at baseline)<br>◊ Collection of week 1 Actiwatch™ data<br>◊ Weigh medications<br>◊ Collection of Diary 1 and Issue of new Diary 2 | ◊ Telephone support from Trial Manager<br>◊ Reminder to start collecting Actiwatch™ data again<br>◊ Reminder to add salt (group A)<br>◊ Reminder to continue taking weekly water samples for hardness testing (Group A) | ◊ SASSAD<br>◊ POEM<br>◊ Utility measures<br>◊ Digital photo of index site<br>◊ Weigh medications<br>◊ Collection of Week 11-12 Actiwatch™ data and Actiwatch™<br>◊ Collection of Diaries 2<br>◊ Issue Diary 3<br>◊ Explanation of cross-over phase | ◊ Remove unit (Group A) within 10 working days of 12 week anniversary of installation<br>◊ Install unit (Group B) within 10 working days of 12 week appointment with RN; retest water hardness.<br>◊ Advice given to reduce soap consumption etc (Group B)<br>◊ Issue water sampling containers and pre-paid envelopes (Group B) | Clinic appointment with Research Nurse to carry out:<br>◊ SASSAD<br>◊ POEM<br>◊ Utility measure<br>◊ Digital photo of index site<br>◊ Collection of Diary 3<br>Visit by Water Engineer to:<br>◊ Remove unit (Group B), if they do not wish to purchase unit, within 10 working days of 4 week anniversary of installation<br>◊ Reinstall unit (Group A) if they wish to purchase unit at first convenient time after 16 week appointment with RN. |

### 9.5 Engineer's visits

Engineers will conduct a screening visit for potential trial participants in order to assess the suitability of their home for the installation of a water softener. A standard checklist will be prepared for this purpose. Any households which are not able to have a unit installed (for whatever reason) will not be enrolled into the study. The pre-recruitment visit will also assess the likely salt requirements of the family, and test water hardness.

If randomised to Arm A, an installation visit will take place as soon as possible following randomisation to treatment (ideally within 10 working days).

For the active treatment group parents will be sent information about how to bypass the unit and asked to do this immediately they return home after their child's 12-week assessment visit. Units will subsequently be removed by the water engineer as soon as possible (ideally within 5 working days) to ensure that the units cannot be turned on again during the final 4-week non-intervention period. If the family chooses to purchase a unit at a reduced price (£446.50) a unit will be reinstalled after they have completed the final 4-week non-intervention period of the study. Families in the active treatment group will send samples of water for hardness testing on a weekly basis throughout the 12 week period when the unit is installed. Those allocated to delayed treatment will receive an active unit at 12 weeks. This will then be removed after week 16 unless the family decides to purchase the unit. Families in the delayed treatment group will send samples of water for hardness testing on a weekly basis from weeks 13 to 16 inclusive.

# 10. STATISTICS

### 10.1 Statistical design

Analyses have been planned in order to place emphasis on objective outcomes that are less likely to be influenced by the potential bias inherent in a single-blind study. Nevertheless, a variety of additional tools are to be used that reflect more closely the disease process throughout the study period. Some of these are relatively objective indicators of disease activity (such as nocturnal movement and treatment application), whilst others reflect subjective concepts (such as self-reported symptoms in the POEM), in order to capture the many health-related dimensions affected by eczema.

The planned analyses should answer the following questions:

1. Does exposure to softened water for 12 weeks improve the symptoms and severity of eczema, compared to standard care?

2. Does softened water improve quality of life for patients and their carers?

3. Are water softeners a cost-effective treatment for children with atopic eczema?

In addition, tertiary analyses will explore the following parameters:

4. How quickly the benefits of softened water become evident.

5. How quickly the benefits of softened water are lost once treatment is stopped.

The main intention-to-treat analysis will be conducted at 12 weeks. An additional per protocol analysis will also be conducted for the primary outcome in order to test the proof of concept.

A sub-group analysis will also be conducted based on the presence or absence of mutations on the gene filaggrin (*see* section 12). Mutations on the filaggrin gene have been associated with dry skin and may therefore be a useful predictor of treatment response.

### 10.2 Sample Size estimate

Sample size estimates are based on other published data relating to the use of SASSAD in patients recruited in secondary care[15], [5]. Based on a minimum clinically relevant difference of 20% in the change in SASSAD score between the 2 groups, and assuming a mean baseline SASSAD score of 20 with a standard deviation in change scores of 10 [15], a sample size of 310 children will provide 90% power, assuming a significance level of 5% and attrition rate of 15% (see planned interventions section). Sample size estimates based on the results of the pilot study, support this sample size estimate.

### 10.3 Primary analyses

◊ Change in disease severity (SASSAD) at 12 weeks compared to baseline, will be assessed using Student's t-test. An adjusted analysis will also be conducted including the stratification variables of eczema severity, age and recruiting centre.

In order to aid the clinical interpretation of these data, the number needed to treat (NNT) assuming a range of improvements in SASSAD scores will also be presented (≥20%, ≥50% and ≥75% improvement).

### 10.4 Secondary analyses

◊ The proportion of time spent moving during the night will be compared using Student's t-test.

◊ The proportion of participants reporting either good or excellent improvement in disease severity on the global assessment scale will be analysed using a chi-squared analysis.

◊ A Student's t-test will be used to assess the difference in the number of grams of topical treatment applied.

◊ POEM scores will be compared at 12 weeks using Student's t-test.

◊ A Student's t-test will be used to assess differences in the number of Totally Controlled Weeks and Well Controlled Weeks throughout the study period.

◊ Mean scores on the Dermatitis Family Impact Scale and Quality of Life scores will be compared using a Student's t-test.

◊ Predictors of response model. Factors to be included in the model will be pre-specified prior to analysis, but will include baseline eczema severity, previous treatment history, water hardness at baseline, prior belief relating to the benefits of softened water, demographic variables and filaggrin status (*see* Section 12).

### 10.5 Tertiary analyses

◊ Changes in SASSAD at 4 weeks will be assessed using Student's t-test.

◊ For the within group analyses, mean change in disease severity (SASSAD) at week 4 (relative to baseline) compared to mean change in disease severity at week 16 (relative to week 12) will be analysed using a paired samples t-test. These analyses will be used to explore speed of onset and possible carry-over effects.

# 11. COST-EFFECTIVENESS ANALYSIS

### 11.1 Objective:

- To assess the cost-effectiveness of installing a water softening unit in the homes of families who have a child with eczema, when compared with usual care.

The cost analysis will compare the overall costs for the intervention to usual care, measuring resource use such as primary care contacts, medication prescribed, secondary care contacts and patient costs. Health and family resource use data will be measured using participant diaries. Resource use will be valued using published unit costs (e.g. Curtis and Netten [16], BNF 2005, and NHS reference costs (http://www.dh.gov.uk/PolicyAndGuidance/OrganisationPolicy/FinanceAndPlanning/NHSReferenceCosts/fs/en)), and patient reported estimates. The costs to the NHS and patient will be reported separately as well as in combination.

The primary measures of effectiveness for cost analyses purposes will be the number of participants who show a ≥50% improvement in SASSAD at 12 weeks compared to baseline. Secondary analyses will be conducted using continuous data from the SASSAD scale; the Dermatitis Family Impact Scale; and the generic measure of health utility as measured on the child version of the EQ-5D (for children aged 3–6 years, the proxy version will be used).

If non-dominance occurs an incremental cost-effectiveness ratio will be produced. Sensitivity analysis will be undertaken to test the robustness of results in the face of any uncertainties or assumptions made in the analysis. In particular, assumptions about the time period over which the difference in costs and difference in benefits are likely to be sustained. Where appropriate the change in health-related quality of life measured on the

EQ-5D will be multiplied by the expected duration of benefits from water softening in order to calculate the Quality Adjusted Life Years (QALYs) of the intervention group compared to the usual care group.

In addition to the cost effectiveness analysis, contingent valuation (CV) methodology will be employed to measure parental willingness to pay for the water softener device as a measure of benefit. CV methodology is now more widely used as a measure of benefit in the health care field [17]. It is an important issue in the context of this study, since it is not clear at this stage who will, or should, pay for the device: the parent or the NHS. Willingness to pay will be asked pre-intervention at the recruitment visit to get an ex-ante hypothetical willingness to pay and again at week 12. At the end of the study, all participants will get the opportunity to purchase the device that they used in the study, thus giving us a measure of parental actual willingness to pay. This will enable us to test whether a hypothetical bias exists in health CV studies in this context.

# 12. ADDITIONAL GENETICS STUDY

### 12.1 The role of filaggrin gene mutations as a predictor of treatment response

Mutations in the gene encoding the skin barrier protein filaggrin have recently been shown to strongly predispose to eczema. Reduced filaggrin activity is associated with an abnormally dry skin and defective skin barrier. It is estimated that up to 50% of children with eczema may carry one or two mutations in the gene encoding filaggrin, which has the gene symbol *FLG* [18]. Individuals carrying one null-allele for filaggrin make only 50% of the normal amount of filaggrin. Often these individuals have a mild form of ichthyosis vulgaris ( a very dry skin) and are at risk for eczema. Individuals who have two null-alleles make no filaggrin and have a more severe form of ichthyosis vulgaris and are at greater risk of eczema[19,20].

In light of this breakthrough in understanding eczema, we have formed collaborative links with the research team that first reported this association (lead by Professor Irwin McLean at the Human Genetics Unit, University of Dundee). We plan to include filaggrin status as a possible predictor of treatment response in the current study. The mechanism of action by which water softeners improve eczema is currently unclear. Nevertheless, it seems intuitive to consider the possibility that a gene associated with dry skin may play an important role in predicting why some people with eczema are more affected by hard water than others.

For the sub-group analysis, study participants will be categorised into two groups according to filaggrin status:
Group 1: *FLG* +/+ (wild type) – control cohort
Group 2: *FLG* +/- (heterozygous for *FLG* null allele) and *FLG* -/- (homozygous for *FLG* null alleles)

This work will refine the phenotypic characteristics of filaggrin-deficient eczema, and will assess whether filaggrin is an important predictive factor in determining treatment

success.

## 12.2 Methodology

To determine filaggrin status it will be necessary to obtain DNA from individuals entering the study. Following written informed consent, participants will be asked to provide a saliva sample at enrolment (or at their 4-week clinic appointment, if more practical). If children are unable to spit into the container, swabs taken from inside the cheek will be used to collect the sample.

Sample containers will be identified using the designated study number and date of birth only. Personal contact details will be kept by the Trial Manager and will not be transferred to the laboratory researchers.

The containers will be shipped to the Human Genetics Unit, University of Dundee. DNA will be extracted by standard techniques and *FLG* genotyping for the common null-alleles will be carried out according to published protocols [19]. Samples will be kept in Dundee for future testing for genes associated with atopic eczema if new techniques become available.

*FLG* genotype status will be recorded and returned to the Centre of Evidence Based Dermatology in Nottingham, again using the study number and date of birth.

## 12.3 Written informed consent

Parents and participants will be offered the possibility of opting out of this part of the study. No participant will be enrolled until informed consent has been gained.

## 12.4 Sample size

The sample size for the main trial is based on the ability to detect at least a 20% difference in disease severity between the two groups (water softener versus usual eczema care). In order to ensure that sufficient power is available for the planned sub group analysis, further sample size calculations have been performed.

Assuming that the presence of at least one mutation in the gene encoding filaggrin results in improved treatment response, a total of 90 children with at least one such mutation would be sufficient to detect a 30% difference between the treatment groups in the primary outcome (disease severity), with 80% power and a significance level of 5% (s.d = 10). Allowing for 20% drop out means that 120 (39% of the children recruited) would need to carry the gene mutation. For 90% power, this figure would be 145 (47%) children. This is in line with previous published findings which suggest that the gene may be present in up to 50% of eczema sufferers[19], although this varies according to ethnic group[21,22].

# 13. TRIAL ADMINISTRATION

## 13.1 Trial personnel

This study will employ a part-time trial manager (60% fte) and 4 part-time research nurses (60% fte). Two MCRN-funded research nurses are also working part-time on the study (London and Lincolnshire). All staff will be supervised by Dr Kim Thomas at the University of Nottingham, although the research nurses employed at the other recruiting centres will report directly to the Principal Investigators for those centres. Water engineering aspects have been sub-contracted by UK WTA to MG Heating, Newport for all installations on the Isle of Wight. Kinetico Ltd, Southampton, are working on behalf of UKWTA, facilitating installations and engineering support on the mainland. Each company has a dedicated water engineer co-ordinator (who is a member of the Trial Management Group) and who will arrange and supervise visits by their own water engineers to participants homes.

In addition, a consumer panel has been convened consisting of 5 service users with experience of living with eczema. Mr David Potter acts as the consumer panel representative on the Trial Steering Committee. Copies of the participant information sheets, symptom diaries and publicity material have been shown to the panel of service users prior to submission for ethical approval. The panel members shared these documents with children with eczema aged 4 and 13 years.

## 13.2 Roles & responsibilities

The Chief Investigator will have overall responsibility for the design, maintenance and delivery of the trial, and will serve as the study guarantor to sponsors, funders and journals.

The Trial Manager will be responsible for all aspects of the day-to-day running of the trial.

The research nurses will be responsible for identifying and recruiting suitable participants, for conducting skin assessments and liaising with the local engineers over visit dates and times (prior to randomisation). Since research nurses are responsible for conducting the blinded outcome assessments, contact after the initial recruitment interview will be kept to a minimum.  Subsequent telephone follow-up will be conducted by the trial manager or study support staff at the co-ordinating centre in Nottingham.

The Clinical Trials Support Unit (University of Nottingham) will provide data management for the trial. This will include a web-based randomisation service (with telephone backup), database design, data entry and central data monitoring.

Professor Andrew Nunn at the MRC Clinical Trials Unit in London will prepare an analysis plan prior to analysis.

### 13.3 Conflicts of interest

None.

### 13.4 Trial Organisation & administration

The trial is funded by the NHS Health Technology Assessment Programme. It is sponsored by the University of Nottingham, and will be managed and co-ordinated from the Centre of Evidence Based Dermatology in Nottingham. Data management will be conducted through the Nottingham Clinical Trials Support Unit. Statistical analysis will be over-seen by Professor Andrew Nunn at the MRC Clinical Trials Unit in London, and conducted by a junior statistician employed at the same trials unit.

Membership of the Trial Steering Committee (TSC) and Trial Management Group (TMG) have been documented at the beginning of this document.

The Trial Steering Committee will meet at least once a year and will provide overall supervision of the trial on behalf of the trial sponsor.

The Trial Management Group will meet more frequently and will be responsible for the day-to-day management of the trial. Members of the TMG will report to the TSC at their meetings.

Since this trial involves the use of a commonly available domestic water softening unit (and does not involve a medicinal product) we do not anticipate the need for a Data Monitoring Committee.

### 13.5 Trial Timetable and Milestones

| | 6 months | 26 months | 4 months | 3 months | 4 months |
|---|---|---|---|---|---|
| | 1 Sep 06<br>to<br>28 Feb 07 | 1 Mar 07 to<br>31 May 09 | 1 Jun 09 to<br>30 Sep 09 | 1 Oct 09 to<br>31 Dec 09 | 1 Jan 10<br>to<br>31 Apr 10 |
| Trial set-up* | | | | | |
| Training** | | | | | |
| Recruitment | | | | | |
| Follow-up | | | | | |
| Data checking | | | | | |
| Database locked | | | | 31 Dec 09 | |
| Analysis / write up | | | | | |

MREC, LREC and R&D approval

* Trial set-up will include the establishment of sponsorship agreement; preparation of trial procedures and CRF's; application for ethics and R&D approvals; purchasing equipment and publishing the protocol. Research nurses employed at each site and the study engineers will be identified during the 6-month set-up period.

** Training of Principal Investigators, Water Engineers and Research Nurses, including awareness of GCP.

### 13.6 Unblinding of participants

This is a single-blind study, in which unblinding of the study participants is not relevant. However, attempts will be made to ensure the continued blinding of the research nurses and the trial statistician. If any of the research nurses feel that blinding may have been compromised at any time, this will be logged accordingly.

## 14. ETHICS

### 14.1 Statement of confidentiality

Any data collected as a result of this trial will be treated as confidential. Participants will be identified by unique reference number and initials wherever possible. It is necessary for participants' name and contact details to be released to the co-ordinating centre, but this will not happen until fully informed consent has been taken.

### 14.2 Data protection

Data will be stored in accordance with the Data Protection Act. Investigators will retain patient records and CRFs in easily retrievable but secure form.

The Chief/Principal investigator will ensure that CRFs and other study documentation relating to their participants are kept in a locked departmental filing cabinet. Completion of, and access to the CRFs will be restricted to those personnel approved by the

Chief/Principal investigator.

# 15. ADVERSE EVENT REPORTING

This trial involves the use of a commonly available domestic water softening unit with provision for mains drinking water during the time when the water softening unit is installed. This being the case, we do not anticipate any adverse events or adverse reactions of any relevance to the trial. As a result adverse event data will not be routinely collected. Events of particular relevance such as plumbing difficulties, floods or difficulties with the units will be logged and reported to the MREC and relevant R&D departments annually.

# 16. TRIAL INSURANCE AND INDEMNITY

### 16.1 Negligent Harm

The usual NHS indemnity arrangements for negligent harm will apply.

### 16.2 Research Liabilities

The sponsor (University of Nottingham) has third-party liability insurance cover in accordance with all local legal requirements.

As a precautionary measure, the investigator, the persons instructed by him and the hospital are included in such cover in respect of work done by them in carrying out this study to the extent that the claims are not covered by their own professional indemnity insurance.

In addition, the study engineers will carry their own 3$^{rd}$ party liability insurance should the installation of the water softening devices result in flood or damage to property.

### 16.3 Non negligent harm

The devices to be used in the study will be covered by product warranty. Other than this, no compensation exists for non-negligent harm.

# 17. PUBLICATION POLICY

During the period of the trial, press releases will be issued from the Centre of Evidence Based Dermatology and will be approved by either the Chief Investigator (Hywel Williams) or the Lead Applicant (Kim Thomas). No party will be entitled to submit any publicity material without prior approval from the co-ordinating centre.

Trial publications and conference presentations will be submitted to the HTA for approval prior to submission to the event organisers or the editors. All publications will acknowledge the support of the HTA in funding this trial, and the support of the water industry.

Neutral or negative results will not constitute a reasonable justification to delay publication.

## 18. TRIAL FINANCES

This trial is funded by the NHS HTA Programme. Appropriate contracts will be established between the University of Nottingham and each of the recruiting sites; with the MRC Clinical Trials Unit, and with the consortium of water treatment companies.

In addition to the monies provided by the NHS HTA programme, representatives from the water industry have agreed to cover the following costs:

- Design, testing and supply of the water softening units.
- Salt supplies
- Testing of the water samples
- Supervision of water engineers

Trial participants will not be paid for taking part in the study, although a standard inconvenience allowance of £5 per visit will be given in the form of gift vouchers. If travel costs are greater than this, trial participants will be given gift vouchers up to a maximum of £10 per clinic visit.

**SIGNATURE PAGE**

**Chief Investigator:** **Professor Hywel Williams**

**Signature: ______________________** **Date: _______________**

**RECRUITING CENTRE**

I confirm that I have read this protocol and agree to conduct the study accordingly.

**Principal Investigator:** ________________________________________

**Recruiting Centre:** ____________________________________

**Signature:** ____________________ **Date:** _____________

## REFERENCES


1. Hoare C, Li Wan Po, A., Williams, H.C. Systematic review of treatments for atopic eczema. *Health Technology Assessment* 2000;**4**(37).
2. McNally NJ, Williams HC, Phillips DR et al. Atopic eczema and domestic water hardness. *Lancet* 1998;**352:**527-531.
3. Miyake Y, Yokoyama T, Yura A, Iki M, Shimizu T. Ecological association of water hardness with prevalence of childhood atopic dermatitis in a Japanese urban area. *Environmental Research* (2004) **94:**33-37
4. Thomas KS, Armstrong, S., Avery, A. et al. Randomised controlled trial of short bursts of a potent topical corticosteroid versus more prolongued use of a mild preparation, for children with mild or moderate atopic eczema. *BMJ* 2002;**324:**768-780.
5. Berth-Jones J. Six area, six sign atopic dermatitis (SASSAD) severity score: a simple system for monitoring disease activity in atopic dermatitis. *British Journal of Dermatology* 1996;**135**(suppl. 48)**:**25-30.
6. Benjamin K, Waterston K, Russell M, Schofield O, Diffey B, Rees JL. The development of an objective method for measuring scratch in children with atopic dermatitis suitable for clinical use. *Journal of the American Academy of Dermatology* 2004;**50**(1)**:**33-40.
7. Bringhurst C, Waterston K, Schofield O, Benjamin K, Rees JL. Measurement of itch using actigraphy in pediatric and adult populations. *Journal of the American Academy of Dermatology* 2004;**51**(6)**:**893-8.
8. Charman CR, Venn AJ, Williams HC. The Patient-Oriented Eczema Measure: Development and Initial Validation of a New Tool for Measuring Atopic Eczema Severity From the Patients' Perspective. *Arch Dermatol* 2004;**140**(12)**:**1513-1519.
9. Langan SM, Thomas KS, Williams HC. What Is Meant by a "Flare" in Atopic Dermatitis?: A Systematic Review and Proposal. *Arch Dermatol* 2006;**142**(9)**:**1190-1196.
10. Lawson V, Lewis-Jones, M.S., Finlay, A.Y., Reid, P., Owens, R.G. The family impact of childhood atopic dermatitis: the dermatitis family impact questionnaire. *British Journal of Dermatology* 1998;**138:**107-113.
11. Lewis-Jones MS, Finlay, A.Y. The children's Dermatology Life Quality Index (CDLQI): initial validation and practical use. *Br J Dematol* 1995;**132:**942-949.
12. Lewis-Jones MS, Finlay AY, Dykes PJ. The Infants' Dermatitis Quality of Life Index. *British Journal of Dermatology* 2001;**144:**104-110.
13. Hennessy S, Kind P. Measuring health status in children: developing and testing a child-friendly version of EQ-5D. EuroQol Scientific Meeting 2002, York: 291-310.
14. Williams HC, Burney, P.G., Hay, R.J., Archer, C.B., Shipley, M.J., Hunter, J.J., Bingham, E.A., Finlay, A.Y., Pembroke, A.C., Graham-Brown, R.A., et al. The UK working party's diagnostic criteria for atopic dermatitis. I. Derivation of a minimum set of discriminators for atopic dermatitis. *British Journal of Dermatology* 1994;**131**(3)**:**383-396.
15. Takwale A, Tan E, Agarwal S, et al. Efficacy and tolerability of borage oil in adults and children with atopic eczema: randomised, double blind, placebo controlled, parallel group trial. *BMJ* 2003;**327**(7428)**:**1385-.
16. PSSRU. Unit costs of health and social care. Canterbury: Personal social services research unit, 2004.
17. Olsen JA, Smith RD. Theory versus practice: a review of 'willingness-to-pay' in health and health care. *Health Econ* 2001;**10**(1)**:**39-52.
18. Palmer CN, Irvine AD, Terron-Kwiatkowski A, et al. Common loss-of-function variants of the epidermal barrier protein filaggrin are a major predisposing factor for atopic dermatitis. *Nat Genet* 2006.
19. Palmer CNA, Irvine AD, Terron-Kwiatkowski A, et al. Common loss-of-function variants of the epidermal barrier protein filaggrin are a major predisposing factor for atopic dermatitis.[see comment]. *Nature Genetics* 2006;**38**(4)**:**441-6.
20. Smith FJD, Irvine AD, Terron-Kwiatkowski A, et al. Loss-of-function mutations in the gene encoding filaggrin cause ichthyosis vulgaris. *Nature Genetics* 2006;**38**(3)**:**337-42.
21. Sinclair C, O'Toole E, Leigh I, et al. Filaggrin mutation is not a genetic risk factor for atopic dermatitis

in the Bangladeshi population. *J Invest Dermatol* 2006;**126** (Suppl 3)**:**s40.
22. Ruether A, Stoll M, Schwarz T, Schreiber S, Folster-Holst R. Filaggrin loss-of-function variant contributes to atopic dermatitis risk in the population of Northern Germany. *British Journal of Dermatology* 2006;**155(**5)**:**1093-1094.

# APPENDIX 1: Recruiting centres

| | |
|---|---|
| **Queen's Medical Centre**<br>Derby Road<br>Nottingham NG7 2UH<br>Main Hospital Tel: 0115 924 9924 | **Principal Investigator:**<br>Professor Hywel Williams<br>Centre of Evidence Based Dermatology<br>University of Nottingham<br>Kings Meadow Campus<br>Lenton Lane<br>Nottingham NG7 2NR; Tel: 0115 846 8619 |
| **Barnet & Chase Farm Hospital**<br>The Ridgeway<br>Enfield<br>Middlesex EN2 8JL<br>Main Hospital Tel: 0845 111 4000 | **Principal Investigator:**<br>Dr Ian Pollock<br>Consultant Paediatrician<br>Dept of Paediatrics<br>Tel: 0208 375 1438 (secretary) |
| **Addenbrooke's Hospital**<br>Cambridge University Hospitals NHS Foundation Trust<br>Hills Road<br>Cambridge<br>Cambridgeshire CB2 2QQ<br>Main Hospital Tel: 01223 245 151 | **Principal Investigator**<br>Dr Nigel Burrows<br>Consultant Dermatologist<br>Dept of Dermatology<br>Tel: 01223 216 501 |
| **The David Hide Asthma and Allergy Research Centre**<br>St Mary's Hospital<br>Isle of Wight Healthcare Trust<br>Newport<br>Isle of Wight PO30 5TG<br>Also:<br>St Mary's Hospital<br>Milton Road<br>Portsmouth PO3 6AD | **Principal Investigator**<br>Professor Tara Dean<br>Reader in Epidemiology/ Director of Research / Associate Head<br>School of Health Sciences and Social Work<br>James Watson Building West<br>2 King Richard I Road<br>University of Portsmouth; Portsmouth, PO1 2FR<br>Tel: (023) 9284 4405; Fax: (023) 9284 5200 |
| **University Hospitals of Leicester NHS Trust**<br>Leicester Royal Infirmary<br>Infirmary Square<br>Leicester LE1 5WW<br>Main Tel: 0116 254 1414 | **Principal Investigator**<br>Dr Robin Graham-Brown<br>Consultant Dermatologist/Hon Senior Lecturer<br>Mills Flat, Victoria Building, Infirmary Square<br>Leicester Royal Infirmary<br>Leicester LE1 5WW<br>Tel: 0116 258 5384 / 0116 258 5162 |
| **United Lincolnshire Hospitals NHS Trust**<br>Lincoln County Hospital<br>Greetwell Road<br>Lincoln LN2 5QY<br>Tel: 01522 512512 | **Principal Investigator**<br>Dr Mansoor Dilnawaz<br>Consultant Dermatologist<br>Dermatology Department<br>Pilgrim Hospital, Sibsey Road<br>Boston PE21 9QS Tel 01205 446436 |
| **Royal London Hospital**<br>Barts & The London NHS Trust<br>Whitechapel, London E1 2AN | **Principal Investigator**<br>Dr Edel O'Toole<br>Consultant Dermatologist<br>Centre for Cutaneous Research<br>Queen Mary University of London<br>London E1 2AT |

# APPENDIX 2: R&D departments

| | |
|---|---|
| **Queen's Medical Centre**<br>Nottingham University Hospitals NHS Trust | R&D Office<br>E11 Curie Court<br>Queens Medical Centre<br>Derby Road<br>Nottingham NG7 2UH<br>Tel: 0115 970 9049 |
| **Barnet & Chase Farm Hospital**<br>Barnet & Chase Farm NHS Trust | Clinical Governance Support Team<br>Thames House<br>Wellhouse Lane<br>Barnet Hospital<br>Middlesex EN5 3DJ<br>Tel: 020 8216 5428 |
| **Addenbrooke's Hospital**<br>Cambridge University Hospitals NHS Foundation Trust | R&D Department<br>Addenbrookes Hospital<br>Box 146<br>Cambridge CB2 2QQ<br>Tel: 01223 217418 |
| **The David Hide Asthma and Allergy Research Centre**<br>Portsmouth Hospitals NHS Trust | R&D Office<br>Gloucester House<br>Queen Alexandra Hospital<br>Cosham PO6 3LY<br>Tel: 023 9228 6236 |
| **Leicester Royal Infirmary**<br>University Hospitals of Leicester NHS Trust | R&D Office<br>Leicester General Hospital<br>Gwendolen Road<br>Leicester LE5 4PW<br>Tel: 0116 258 4109 |
| **The Royal London Hospital**<br>Barts & The London NHS Trust | Joint R&D Office<br>24-26 Walden Street<br>Whitechapel<br>London E1 2AN |

# Softened Water Eczema Trial (SWET)

# Statistical Analysis Plan

**Author: Caroline Gilbert**

**TABLE OF CONTENTS**

**PAGE**

ABBREVIATIONS ........ 3

1. INTRODUCTION ........ 4

2. TRIAL OBJECTIVES ........ 4

3. ENDPOINTS ........ 4
   3.1. Primary Endpoint ........ 4
   3.2. Secondary Endpoints ........ 5
   3.3. Tertiary Endpoints ........ 6

4. SAMPLE SIZE ........ 6

5. ITT ANALYSIS AND MULTIPLICITY ........ 6

6. STRATA AND COVARIATES ........ 7
   6.1. Stratification variables ........ 7
   6.2. Other covariates ........ 7

7. PLANNED SUBGROUP ANALYSES ........ 8

8. DATA HANDLING ........ 8
   8.1. Missing data ........ 8
   8.2. Partial dates ........ 8
   8.3. Derived Variables ........ 9
   8.4. Bias ........ 9
   8.5. Data quality ........ 10

9. PARTICIPANT CHARACTERISTICS AND COMPLIANCE ........ 10
   9.1. Demographic and Baseline Characteristics ........ 10
   9.2. Compliance ........ 10
   9.3. Withdrawals ........ 10

10. EFFICACY ........ 10
    10.1. Primary Efficacy Analyses ........ 10
    10.2. Secondary Efficacy Analyses ........ 11
    10.3. Tertiary efficacy analyses ........ 11

11. ADDITIONAL ANALYSES ........ 11

12. REFERENCES ........ 12

13. SIGNATURE PAGE ........ 13

## ABBREVIATIONS

| | |
|---|---|
| CTSU | Clinical Trials Support Unit |
| CV | Contingent Valuation |
| DFI | Dematitis Family Impact |
| HTA | Health Technology Assessment |
| ITT | Intention To Treat |
| NIHR | National Institute for Health Research |
| POEM | Patient Oriented Eczema Measure |
| QALY | Quality Adjusted Life Years |
| SASSAD | Six Area Six Sign Atopic Dermatitis Score |
| SWET | Softened Water Eczema Trial |
| TCW | Totally Controlled Weeks |
| TMG | Trial Management Group |
| TSC | Trial Steering Commitee |
| WCW | Well Controlled Weeks |

## 1. INTRODUCTION

This analysis plan details the planned statistical analyses for the Softened Water Eczema Trial (SWET).

This is a single-blind, parallel group, randomised controlled trial of 12-week duration, followed by a 4-week cross-over period. The objective is to assess whether the installation of an ion-exchange water softener improves eczema in children, and if so to establish the likely cost and cost-effectiveness of the intervention.

Participants are randomised to one of two groups on a 1:1 basis. The first group have a water softener installed the first 12 weeks of the trial followed by a 4 week washout period. The second group, the delayed treatment group, have the water softener installed for four weeks from week 12 onwards.

| | STUDY PERIOD = 16 weeks | |
|---|---|---|
| | 0 to 12 weeks | 12 to 16 weeks |
| Group A | Usual eczema care + water softener installed (n = 155) | Unit removed |
| Group B | Usual eczema care + delayed installation (n = 155) | Unit installed |

Throughout this document Group A will be referred to as the immediate installation group, and Group B as the delayed treatment group.

The analyses described in this document will be performed by the designated statistician at the MRC Clinical Trials Unit. All data will be analysed using Stata Version 10.1.

## 2. TRIAL OBJECTIVES

There are two key objectives for the trial:

1. To assess whether the installation of an ion-exchange water softener improves eczema in children
2. If so, to establish the likely cost and cost-effectiveness of this intervention

## 3. ENDPOINTS

Due to the nature of the intervention this study is being conducted as a single blind trial. For this reason objective, and validated outcome measures have been used (Schmitt, 2007).

### 3.1. Primary Endpoint

Participants with moderate to severe eczema will be randomised to one of the two groups described previously.

The primary endpoint is the difference between the immediate installation and delayed intervention groups with regard to mean change in disease severity (Six Area Six Sign Atopic Dermatitis Score – SASSAD) at 12 weeks compared to baseline.

### 3.2. Secondary Endpoints

1. The difference between baseline and week 12 of the proportion of time spent moving during the night. Movement will be captured for periods of one week at weeks 1 and 12, and will be measured using accelerometers (Actiwatch). These units are worn by the child in the same way as a wrist watch. Due to possible differences between the units every effort was made to ensure the children always used the same unit and where this was not the case this has been documented. A sensitivity analysis will be performed excluding those who did not use the same unit throughout the trial.

2. Difference in the proportion of children who report either a good or excellent improvement in eczema severity at 12 weeks (using a 5-point Likert scale). As these data were not collected[1], responders will be grouped into three groups; those who report a reasonable (≤20%), good (>20% and ≤50%) or excellent (>50%) improvement in SASSAD score at 12 weeks.

3. Amount of topical corticosteroid / calcineurin inhibitors used during the 12 week study period. This information is captured by weighing the medication at each visit and then summarised according to steroid strength to give the total amount used from baseline to week 12. Nurses will also be providing an indication of the level of accuracy of any estimates. A sensitivity analysis will be performed excluding those for whom the nurse is not confident about the amount of medication used.

4. Difference in Patient Oriented Eczema Measure (POEM). This scale is a well validated tool that has been developed to capture symptoms of importance to patients (rather than objective signs that are used in traditional severity scales, such as SASSAD).

5. Difference in the number of totally controlled weeks (TCW) and well-controlled weeks (WCW) based on the number of days with eczema symptoms and the number of days that topical treatment is applied up to primary endpoint at 12 weeks. This will be derived from the symptom diaries.

6. Difference in the mean change from baseline in the Dermatitis Family Impact (DFI) questionnaire at 12 weeks.

---

[1] Omitted from CRFs to avoid unblinding of research nurses, and also on reflection this measure would be too open to bias in a single-blind study. Therefore improvement based on improvement in SASSAD score is preferable.

### 3.3. Tertiary Endpoints

If a beneficial effect is found the following tertiary analyses will be performed.

1) Speed of onset of benefit will be analysed in three ways. Firstly, the difference in the mean change in disease severity (SASSAD) at 4 weeks compared to baseline. Secondly, the final 4 week period (weeks 12 to 16) will be examined for the delayed treatment group. Finally the daily bother scores will be examined for the first 4 weeks of the trial for both groups.

2) Likely carry over effect will be examined using the full 16 week trial period for the immediate installation group. This information will be useful in planning the design of future trials in this area.

3) Predictors of response model, including baseline factors (see section 6.2)

## 4. SAMPLE SIZE

Sample size estimates are based on other published data relating to the use of SASSAD in patients recruited in secondary care. Based on a minimum clinically relevant difference of 20% in the change in SASSAD score between the 2 groups, and assuming a mean baseline SASSAD score of 20 with a standard deviation in change scores of 10, a sample size of 310 children will provide 90% power, with a significance level of 5% allowing for an attrition rate of 15%.

## 5. ITT ANALYSIS AND MULTIPLICITY

The SWET trial will be analysed as intent-to-treat (ITT) at week 12.

The ITT population will consist of all randomised participants with evaluable data. This will be the primary population used for the main analysis, which will use the randomised treatment allocation rather than actual treatment received. An additional sensitivity analysis will be performed excluding outliers.

Primary inference will be based on the primary endpoint analysis of the ITT population. Significance will be at the 5% level.

A secondary, per protocol analysis of the primary endpoint will be performed excluding the following participants:

- Those who were randomised into the study, but who failed to receive their allocated treatment.
- Those who are deemed to be major protocol violators as determined by the Protocol Violators Group (including independent members) review of the protocol deviation log.

Criteria for protocol violators are as follows:

- Missing SASSAD score at week 12
- Group A: exposed to fully softened water for <75% of the time their home has an active water softener in place (i.e. sleeping at home + unit fully working for <75% of the time their home has an installation)

- Group A: Participant away from home or with partially functioning water softener of >2 days/week for each of the 4 weeks prior to the primary outcome measure
- Group B: Participant away from home for > 2 days/week for each of the 4 weeks prior to the primary outcome measure
- Unblinding of research nurse prior to primary outcome measurement
- Starting new treatment prior to primary outcome measurement will be examined on a case-by-case basis to determine if they comprise a protocol violator.

Baseline characteristics (as described in section 6.2) will be summarised and if any major imbalance exists the analyses will be adjusted to account for this.

Analyses of all secondary endpoints and adjusted analyses will be considered supportive to the primary analysis so no adjustments for multiple comparisons will be made.

## 6. STRATA AND COVARIATES

### 6.1. Stratification variables

Randomisation is stratified by the following:

- Disease severity (baseline SASSAD ≥10 to ≤ 20, or SASSAD score >20)
- Recruiting centre

### 6.2. Other covariates

In addition to the stratification variables, other covariates to be considered in a predictors of response model are:

- Age (continuous)
- Previous treatment history (see section 8.3)
- Water hardness at baseline (WHO classification, continuous).
- Sex
- Ethnicity
- Filaggrin status
- Income
- Time away from home (based on a cut off of >21 days).

In addition, the following baseline characteristics will be summarised:

- Washing powder

- Fabric softener
- Bathing/showering frequency
- Swimming frequency
- Home ownership
- Allergy

## 7. PLANNED SUBGROUP ANALYSES

A sub-group analysis will be conducted based on the presence or absence of mutations on the filaggrin gene (collected using spit samples). Mutations on the filaggrin gene have been associated with dry skin and may therefore be a useful predictor of treatment success. The planned subgroup analysis will only be performed on the primary outcome of change in SASSAD score. The p-value for the interaction will be reported.

Assuming that the presence of at least one mutation in the gene encoding filaggrin results in improved treatment response, a total of 90 children with at least one such mutation would be sufficient to detect a 30% difference between the treatment groups in the primary outcome, with 80% power, 5% significance (2 sided) and a standard deviation of 10. Allowing for a 20% drop out means that 120 children would need to carry the gene mutation. For 90% power this figure would be 145.

## 8. DATA HANDLING

### 8.1. Missing data

The primary outcome is collected by nurses at study visits so missing items are not expected. Other missing data items such as age, sex, etc. will be queried so that there are no missing data for these variables.

Missing baseline water hardness score will be replaced with published water hardness data for that postcode.

For each endpoint the number with a missing outcome for each treatment group will be reported with reasons given where available.

If less than 5% of data are missing for the primary endpoint only participants with complete data will be included in the full ITT primary analysis. An additional sensitivity analysis will be conducted replacing missing data with the maximum value at baseline and week 4 (where this exists). If more than 5% of data are missing for the primary endpoint then more complex multiple imputation techniques will be used to handle the missing values. Secondary endpoint analyses will be analysed using a complete case analysis as these are only considered supportive of the primary endpoint analysis.

### 8.2. Partial dates

Missing months will be taken as June and missing days will be taken as the 15$^{th}$ day of the month.

## 8.3. Derived Variables

SASSAD scores will be computed prior to data entry. This will be checked using the individual components within the analysis files.

The proportion of time spent moving during the night will be obtained using accelerometers. The average of the first three nights of usable data at week 0 and the last three nights of usable data at week 12 will be used. Usable data is defined as values greater than 5% and less than 95% of the night spent moving to remove outliers. If there are less than three usable nights data, then this variable will be considered missing.

Patient Orientated Eczema Measure (POEM) scores will be calculated as a score between 0 and 28 based on seven symptoms. Parents are asked to state the number of days in the last week their child has been affected by each symptom. These are scored as follows; no days=0, 1-2 days=1, 3-4 days=2, 5-6 days=3 and everyday=4. The POEM score is the calculated as the sum of these 7 individual scores.

Totally Controlled Weeks (TCW) will be defined as zero days with an eczema bother score greater than 4 and zero days where "stepping up" of treatment was needed. Well-Controlled Weeks (WCW) will be defined as two days or less with an eczema bother score greater than 4 and two days or less days where "stepping up" of treatment was needed. The eczema score is recorded by the parent each day using a symptom diary and is based on how much bother the child's eczema has been that day. The score is on a scale from 0 to 10 where 0 equals no bother at all and 10 equals the most bother you can imagine. The definition of "stepping up" varies from child to child and is documented in the symptom diary for the parent to refer to after discussion with the nurse at the beginning of the trial.

Dermatitis Family Impact (DFI) scores will be calculated as a score between 0 and 30 based on ten questions. Parents are asked to state how much the child's skin problem has affected the family over the last week These are scored as follows; not at all=0, a little=1, a lot=2 and very much=3. The DFI score is the calculated as the sum of these 10 individual scores.

Age (in years and months) will be calculated at randomisation.

Previous treatment history will be assessed using treatment reported at enrolment. This will be grouped into low strength (mild and moderate topical steroids) and high strength (potent and very potent steroids and mild and moderate calcineurin inhibitors). Participants will be classed as a low strength user if they only use mild and moderate topical steroids, and a high strength user if the use potent or very potent topical steroids or mild or moderate calcineurin inhibitors (even if they also use mild or moderate topical steroids).

## 8.4. Bias

The research nurses are blinded to treatment allocation throughout the study period. If any of the nurses feel that this blinding may have been compromised, details are logged centrally with the Trial Manager. A Sensitivity analysis will be conducted excluding those participants for whom the research nurse became unblinded to treatment allocation

during the trial. Integrity of information bias will be assessed using clinical photographs of a target lesion.

### 8.5. Data quality

Data queries will be resolved at data entry using a query form. To minimise errors, all primary outcome data will be verified by a data entry clerk who did not originally enter the data. A 10% sample of all other data will be checked for accuracy.

## 9. PARTICIPANT CHARACTERISTICS AND COMPLIANCE

### 9.1. Demographic and Baseline Characteristics

Demographic, disease severity, filaggrin status and other baseline characteristics will be cross-tabulated against randomised treatment allocation to check for appropriate balance. If substantial imbalance exists an additional adjusted analysis will be performed.

### 9.2. Compliance

Absence from the home will be used as the measure of compliance (recorded on the symptom diary).

A log of all protocol deviations will be kept by the Trial Manager which will be reviewed by the protocol violations committee at the end of the trial in order to assign the events as major or minor protocol deviations. Participants with major deviations will be excluded from the per protocol analysis.

### 9.3. Withdrawals

If participants choose to withdraw from the study, any units that have been installed will be removed as soon as is practicably possible. Participants will be asked to complete an end of study questionnaire at this time and diaries collected.

The number of participants who withdraw from the study with the reasons for withdrawal will be summarised by randomised treatment allocation.

## 10. EFFICACY

### 10.1. Primary Efficacy Analyses

The primary analysis will be the comparison of the change from baseline in SASSAD scores at 12 weeks between the two intervention groups using a t-test, including a p-value and confidence interval. This will also be expressed as the number needed to treat for a 20% reduction in SASSAD score at week 12 and a 50% reduction.

## 10.2. Secondary Efficacy Analyses

1) The difference between baseline and week 12 of the proportion of time spent moving during the night will be compared across intervention groups using a t-test. The average of the first three nights of evaluable data at week 0, and the last three nights at week 12 will be used.

2) The proportion of children who report a reasonable (≤20%), good (>20% and ≤50%) or excellent (>50%) improvement in SASSAD score at 12 weeks, will be compared using a chi-squared test.

3) Amount of topical corticosteroid / calcineurin inhibitors used during the 12 week study period. This will be captured by weighing the medication at each visit. This will then be summarised by the nurses at week 12, including a measure of the degree of accuracy of any estimates. These will be spilt into two groups, low strength consisting of mild and moderate topical steroids, and high strength consisting of potent and very potent topical steroids and mild and moderate calcineurin inhibitors. The difference in the amount (in grams) of each of the two groups will be assessed using t-tests.

4) The difference in the change from baseline to week 4 and baseline to week 12 Patient Oriented Eczema Measure (POEM) scores will be compared using t-tests.

5) Difference in the number of well controlled weeks (WCW) as defined in section 8.3, will be analysed using a t-test. The number of totally controlled weeks (which are a subset of the WCW), will also be summarised.

6) Difference in the mean change from baseline in the Dermatitis Family Impact (DFI) questionnaire at 12 weeks, will be analysed using a t-test.

## 10.3. Tertiary efficacy analyses

1) Speed of onset of benefit will be analysed in three ways. Firstly, the difference in the mean change in disease severity (SASSAD) at 4 weeks compared to baseline will be analysed using a t-test. Secondly, the final 4 week period (weeks 12 to 16) will be examined for the delayed treatment group using a single sample t-test. Finally daily bother scores from the daily symptom diaries for the first 4 weeks of the trial will be plotted for both groups.

2) Likely carry over effect will be examined graphically using the daily bother scores during the 16 week trial period for the immediate installation group. This information will be useful in planning the design of future trials in this area.

3) A responder will be defined as a 20% or 50% decrease in SASSAD score. Two logistic regression models will be used to investigate predictors of response as listed in section 6.2.

# 11. ADDITIONAL ANALYSES

Descriptive statistics will be used to summarise the data from the end-of-trial questionnaire added part way through the trial.

## 12. REFERENCES

Schmitt J, Langan S, Williams H. What are the best outcome measurements for atopic eczema? A systematic review. *J Allergy Clin Immunol* 2007, 120:1389-1398

## 13. SIGNATURE PAGE

**Chief Investigator: Professor Hywel Williams**

**Signature: ___________________________ Date: ________________**

**Designated Statistician: Caroline Gilbert**

**Signature: ___________________________ Date: ________________**

**Trial Steering Committee Chair: Dr David Paige**

**Signature: ___________________________ Date: ________________**

**Lead Applicant: Dr Kim Thomas**

**Signature: ___________________________ Date: ________________**
